# Mechanistic Modelling of Transdermal Delivery Dependence on Drug Properties

**DOI:** 10.1007/s11095-026-04027-1

**Published:** 2026-02-20

**Authors:** Neil Benbrook, Wenbo Zhan

**Affiliations:** School of Engineering, University of Aberdeen, Aberdeen, AB24 3UE UK

**Keywords:** drug transport, mechanistic analysis, microneedle, transdermal delivery

## Abstract

**Purpose and objective:**

Microneedles have emerged as a promising platform for transdermal drug delivery, offering high patient compliance, ease of use, and minimal invasiveness. Despite extensive research on microneedle design and fabrication, the influence of intrinsic drug properties on delivery performance remains insufficiently understood. This study is aimed to determine the individual effects of key transport properties of the loaded drug on delivery outcomes across different skin layers and the systemic circulation.

**Methods:**

A multiphysics model is employed to characterise transdermal drug delivery via microneedles, based on a multilayer skin model that incorporates realistic anatomical structures and dimensions. Nine key drug-related parameters are investigated, including drug diffusivity in the microneedle and skin tissues, partition coefficients between the tissue and microneedle, between the cell membrane and interstitial space, and between the cell interior and interstitial space, as well as the protein binding coefficient, transvascular permeability, elimination rate in the skin tissue, and plasma clearance.

**Results:**

The simulations reveal distinct responses of drug delivery performance in each skin layer and in the blood circulation to variations in each property, with optimal values existing depending on the location of the therapeutic target within the skin.

**Conclusions:**

The findings provide mechanistic insights into the interplay between drug physicochemical characteristics and transdermal transport dynamics, offering valuable guidance for rational drug selection, formulation design, and the development of microneedle-based therapeutics.

## Introduction

Transdermal drug delivery has attracted extensive attention for the treatment of several disorders owing to its distinct advantages, including superior patient compliance, non-invasiveness, and convenience of administration [1]. This approach has been successfully applied to manage several conditions, including chronic diseases [2–5] and cancers [6–8]. In conventional transdermal systems, drugs are typically administered on the skin surface. However, due to the complex anatomical structure of the skin, drug molecules must successively permeate the epidermis to reach the papillary dermis, where the vascular and lymphatic networks are located [9]. Once delivered into the papillary dermis, the drugs can transfer across the blood capillary walls into the tissue blood and subsequently enter systemic circulation via blood perfusion. The remaining fraction of the drug may be absorbed by lymphatic vessels or diffuse further into the deeper reticular dermis.

Despite these potential advantages, the efficiency of transdermal delivery is considerably hindered by the stratum corneum, a lipid-protein bilayer that serves as the principal protective barrier of the skin [10]. This barrier, composed of corneocytes embedded within densely packed lipid matrices, effectively prevents the permeation of most external substances. Therefore, overcoming the stratum corneum remains a critical determinant for enhancing the effectiveness of transdermal delivery. Microneedles have been engineered as a promising technology to overcome this limitation. By generating micro-scale pathways across the stratum corneum, microneedles allow drugs to be released directly into the viable skin layers, thereby enhancing penetration and absorption [11]. A broad range of therapeutic agents has been successfully incorporated into microneedles for targeted delivery [12–18]. However, the subsequent transport, distribution, and accumulation of drugs within the skin and systemic circulation are governed by multiple coupled physiological and physicochemical processes. These processes are highly dependent on both tissue characteristics and the intrinsic properties of individual drugs. Hence, understanding how specific drug properties influence delivery performance is essential for optimising therapeutic efficacy.

Evaluation of these effects, however, often requires a controlled and reproducible tissue environment, which is difficult and cost-prohibitive to achieve through clinical or *in vivo* studies. Mathematical modelling provides an effective and economical alternative. By employing governing equations that describe interrelated drug transport processes, mathematical models allow systematic exploration of key parameters under well-controlled conditions [19]. Several models have been developed to elucidate different aspects of microneedle-mediated delivery. For instance, Prakash *et al.* investigated the influence of microneedle geometry and delivery regimens on drug transport using hollow microneedles [20]. Bhuimali *et al.* analysed the effects of drug metabolism and tissue retention for verapamil delivery [21, 22], while Yadav developed a model [23] for swellable microneedles, highlighting the roles of skin viscoelasticity, microneedle insertion depth, and initial drug loading [24]. Tian and colleagues further simulated ultrasound-triggered release from microneedles [25]. Nevertheless, a comprehensive analysis focusing specifically on the role of physicochemical properties of plain drugs remains lacking.

This study employs a mathematical model to investigate how a range of physicochemical properties of plain drugs affect transdermal delivery using microneedles. The model incorporates a multilayered skin structure that reflects realistic anatomical features and captures the major physiological and physicochemical processes, including interstitial fluid flow influenced by trans-epidermal water loss, drug transfer within and among microneedles, skin tissues and circulatory systems, reversible protein binding, cellular uptake, metabolic elimination, physical degradation, and plasma clearance. The delivery outcomes are quantified in terms of average drug concentration and exposure in each skin layer and systemic compartment.

## Methods and Materials

### Mathematical Model

The viable skin tissues can be considered porous media where the interstitial fluid perfuses in the gaps between cells. The flow of the interstitial fluid can thereby be modelled using the mass equation and Darcy’s law, as

1 $$\nabla \bullet {\mathbf{u}}_{\mathrm{ISF}}=\left\{\begin{array}{ll}{F}_{\mathrm{BL}}-{F}_{\mathrm{LY}}& \text{in papillary dermis}\\ 0& \text{in viable epidermis},\text{ reticular dermis}\end{array}\right.$$

2 $${\mathbf{u}}_{\mathrm{ISF}}=-\frac{{\kappa }_{\mathrm{TIS}}}{{\mu }_{\mathrm{ISF}}}\nabla {p}_{\mathrm{ISF}}$$

where ${\mu }_{\mathrm{ISF}}$ is the interstitial fluid’s viscosity. ${p}_{\mathrm{ISF}}$ and ${\mathbf{u}}_{\mathrm{ISF}}$ are the pressure and velocity of the interstitial fluid flow, respectively. ${F}_{\mathrm{BL}}$ governs the fluid leakage from the blood lumen to the tissue, following Starling’s law, as

3 $${F}_{\mathrm{BL}}={L}_{\mathrm{BV}}\frac{{S}_{\mathrm{BV}}}{{V}_{\mathrm{TIS}}}\left[{p}_{\mathrm{BL}}-{p}_{\mathrm{ISF}}-{\sigma }_{\mathrm{BP}}\left({\pi }_{\mathrm{BL}}-{\pi }_{\mathrm{ISF}}\right)\right]$$

where ${L}_{\mathrm{BV}}$ denotes the hydraulic conductivity of the blood capillary wall, indicating the ease with which fluid passes through it under a pressure gradient. ${S}_{\mathrm{BV}}$ is the surface area of the blood capillary wall, and ${V}_{\mathrm{TIS}}$ is the tissue volume. ${p}_{\mathrm{BL}}$ presents the pressure of the blood in the skin tissues. ${\sigma }_{\mathrm{BP}}$ is the reflection coefficient for plasma proteins. ${\pi }_{\mathrm{BL}}$ and ${\pi }_{\mathrm{ISF}}$ are the osmotic pressure of the blood and interstitial fluid, respectively. ${F}_{\mathrm{LY}}$ describes the fluid leakage rate from the papillary dermis to the lymphatics, defined as

4 $${F}_{\mathrm{LY}}={L}_{\mathrm{LV}}\frac{{S}_{\mathrm{LV}}}{{V}_{\mathrm{TIS}}}\left({p}_{\mathrm{ISF}}-{p}_{\mathrm{LY}}\right)$$

where ${L}_{\mathrm{LV}}$ represents the hydraulic conductivity of lymphatic vessel walls, and ${S}_{\mathrm{LV}}$ is the surface area. ${p}_{\mathrm{LY}}$ is the lymphatic pressure.

**Fig. 1:**
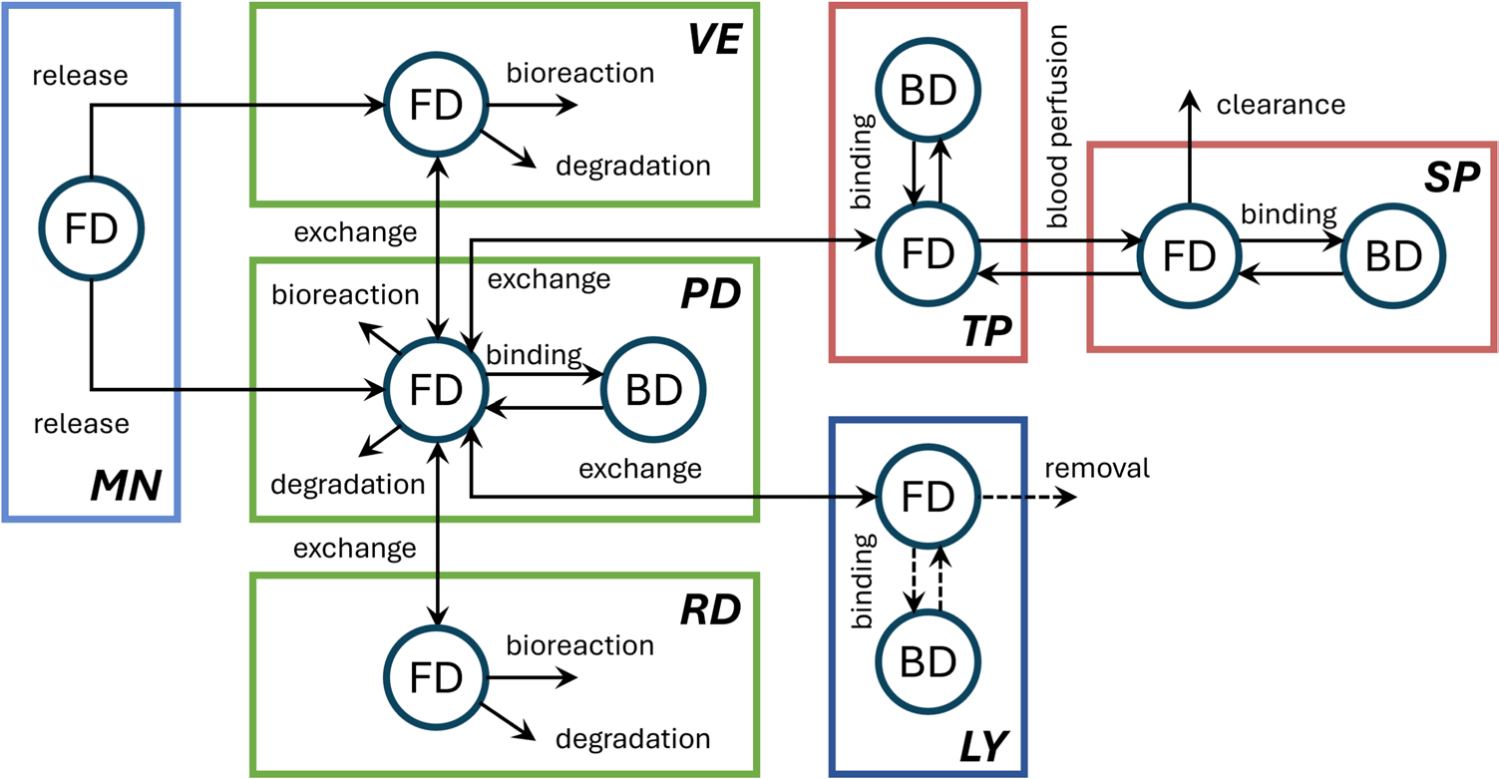
Drug transport in microneedle-assisted transdermal delivery of plain drugs across skin layers and into the circulating systems. The lymphatic system is considered a sink term; therefore, drug transport processes within this system are not explicitly represented in the model and are indicated by dashed lines. Since proteins involved in binding processes, e.g. albumin, are primarily supplied by the circulatory system, this interaction is considered only within the papillary dermis. The biochemical reactions between the drug and other proteins and biomolecules that may reduce the drug concentration are accounted for through metabolic processes. Abbreviations: FD, free drug; BD, protein-bound drug; MN, microneedle; VE, viable epidermis; PD, papillary dermis; RD, reticular dermis; TP, tissue blood; SP, systemic blood; LY, lymphatic system.

Figure 1 illustrates the drug transport processes among the microneedle, skin tissues, and the blood and lymphatic circulatory systems. Drugs are released from the microneedle into the skin tissues, where free drugs can undergo bidirectional transfer between two adjacent skin layers, and simultaneously experience elimination through physical degradation and biochemical reactions. Drugs can also bind to proteins, which are extravasated from blood capillaries. Free drugs may also transfer to the circulatory systems at the papillary dermis. Due to blood perfusion, drugs in the tissue blood can further travel into the systemic blood, where they are subject to clearance processes.

Drugs are assumed to be well encapsulated within the microneedles (MNs) without undergoing elimination or irreversible binding to the microneedle materials. Therefore, its transport in the microneedles is governed as the diffusion process, as

5 $$\frac{\partial {C}_{\mathrm{FD}}}{\partial t}=\nabla \bullet \left({D}_{\mathrm{MN}}\nabla {C}_{\mathrm{FD}}\right)$$

in which ${D}_{\mathrm{FD},\text{ MN}}$ denotes the diffusivity of free drugs in the microneedles.

The skin tissue can be conceptually partitioned into three compartments, including the interstitial space (IS), cell membrane (CM), and cell interior (CI), respectively. Drugs among the tissue compartments can present in their free form (FD) and bind to proteins (BD). Therefore, following the principle of mass conservation, the drug concentrations can be calculated by

6 $$\begin{array}{c}{C}_{\mathrm{FD}}={\upsilon }_{\mathrm{IS}}{C}_{\mathrm{FD},\mathrm{IS}}+{\upsilon }_{\mathrm{CM}}{C}_{\mathrm{FD},\mathrm{CM}}+{\upsilon }_{\mathrm{CI}}{C}_{\mathrm{FD},\mathrm{CI}}\\ {C}_{\mathrm{BD}}={\upsilon }_{\mathrm{IS}}{C}_{\mathrm{BD},\mathrm{IS}}+{\upsilon }_{\mathrm{CM}}{C}_{\mathrm{BD},\mathrm{CM}}+{\upsilon }_{\mathrm{CI}}{C}_{\mathrm{BD},\mathrm{CI}}\end{array}$$

where ${\upsilon }_{\mathrm{IS}}$, ${\upsilon }_{\mathrm{CM}}$, and ${\upsilon }_{\mathrm{CI}}$ are the volume fractions of the interstitial space, cell membrane, and cell interior, respectively; their sum is equal to 1.0. It is assumed that no drugs are bound to protein on cell membrane, so that the drug concentration in this compartment is set to zero [26]. Drug transfer in the skin tissue through diffusion driven by concentration gradient, convection with the interstitial fluid flow, elimination due to enzyme and non-enzyme involved reactions, and binding to proteins. Moreover, the blood capillaries and lymphatic systems in the papillary dermis can also contribute. Therefore, the free drug concentration can be written as

7 $$\frac{\partial {C}_{\mathrm{FD}}}{\partial t}={\nabla }^{2}\left({D}_{\mathrm{FD},\mathrm{IS}}{\upsilon }_{\mathrm{IS}}{C}_{\mathrm{FD},\mathrm{IS}}\right)-\nabla \bullet \left({\mathbf{u}}_{\mathrm{ISF}}{\upsilon }_{\mathrm{IS}}{C}_{\mathrm{FD},\mathrm{IS}}\right)-{k}_{\mathrm{re}}{\upsilon }_{\mathrm{IS}}{C}_{\mathrm{FD},\mathrm{IS}}-{P}_{\mathrm{FD}}\frac{{S}_{\mathrm{BV}}}{{V}_{\mathrm{TIS}}}\left({C}_{\mathrm{FD},\mathrm{IS}}-{C}_{\mathrm{FD},\mathrm{TP}}\right)-{F}_{\mathrm{LY}}{\upsilon }_{\mathrm{IS}}{C}_{\mathrm{FD},\mathrm{IS}}-{k}_{\mathrm{re}}{\upsilon }_{\mathrm{CI}}{C}_{\mathrm{FD},\mathrm{CI}}-\frac{\partial {C}_{\mathrm{BD}}}{\partial t}$$

where ${D}_{\mathrm{FD},\mathrm{IS}}$ is the free drug’s diffusivity in the tissue. ${k}_{\mathrm{re}}$ is the drug elimination rate due to reactions. ${P}_{\mathrm{FD}}$ refers to the transvascular permeability of free drugs. ${C}_{\mathrm{FD},\mathrm{TP}}$ is the free drug’ concentration in the tissue blood.

Since this binding of drug to proteins is a binary process occurring on a much smaller time scale compared to drug transport, it is assumed that the concentration of protein-bound drugs is linearly correlated to the concentration of free drugs in both the interstitial space and cell interior, as ${K}_{\mathrm{IS}}={C}_{\mathrm{BD},\mathrm{IS}}/{C}_{\mathrm{FD},\mathrm{IS}}$ and ${K}_{\mathrm{CI}}={C}_{\mathrm{BD},\mathrm{CI}}/{C}_{\mathrm{FD},\mathrm{CI}}$ [26]. Dynamic equilibrium of drug concentrations can be established between the cell membrane and the interstitial space, and between the cell interior and the interstitial, as ${P}_{\mathrm{CM}-\mathrm{IS}}={C}_{\mathrm{FD},\mathrm{CM}}/{C}_{\mathrm{FD},\mathrm{IS}}$ and ${P}_{\mathrm{CI}-\mathrm{IS}}={C}_{\mathrm{FD},\mathrm{CI}}/{C}_{\mathrm{FD},\mathrm{IS}}$ [26]. Therefore, Eq. (7) can be simplified as,

8 $$\frac{\partial {C}_{\mathrm{FD},\mathrm{IS}}}{\partial t}={D}_{\mathrm{FD},\mathrm{IS}}^{*}{\nabla }^{2}{C}_{\mathrm{FD},\mathrm{IS}}-{\mathbf{u}}_{\mathrm{ISF}}^{*}\bullet \nabla {C}_{\mathrm{FD},\mathrm{IS}}-{k}_{\mathrm{bl}}^{*}\left({C}_{\mathrm{FD},\mathrm{IS}}-{C}_{\mathrm{FD},\mathrm{TP}}\right)-{k}_{\mathrm{elm}}^{*}{C}_{\mathrm{FD},\mathrm{IS}}$$

where ${D}_{\mathrm{FD},\mathrm{IS}}^{*}={D}_{\mathrm{FD},\mathrm{IS}}{\upsilon }_{\mathrm{IS}}/m$ is the apparent diffusivity of free drugs in the interstitial space. ${\mathbf{u}}_{\mathrm{ISF}}^{*}={\mathbf{u}}_{\mathrm{ISF}}{\upsilon }_{\mathrm{IS}}/m$ represents the apparent interstitial fluid velocity. ${k}_{\mathrm{bl}}^{*}={P}_{\mathrm{FD}}\frac{{S}_{\mathrm{BV}}}{{V}_{\mathrm{TIS}}}/m$ is the apparent rate of blood drainage. ${k}_{\mathrm{elm}}^{*}=\left[{\upsilon }_{\mathrm{IS}}\left({F}_{\mathrm{BL}}-{F}_{\mathrm{LY}}\right)+{k}_{\mathrm{re}}\left({\upsilon }_{\mathrm{IS}}+{P}_{\mathrm{CI}-\mathrm{IS}}{\upsilon }_{\mathrm{CI}}\right)+{F}_{\mathrm{LY}}{\upsilon }_{\mathrm{IS}}\right]/m$ is the apparent elimination rate. $m={\upsilon }_{\mathrm{IS}}\left(1+{K}_{\mathrm{IS}}\right)+{\upsilon }_{\mathrm{CI}}{P}_{\mathrm{CI}-\mathrm{IS}}\left(1+{K}_{\mathrm{CI}}\right)+\left(1-{\upsilon }_{\mathrm{IS}}-{\upsilon }_{\mathrm{CI}}\right){P}_{\mathrm{CM}-\mathrm{IS}}$ is determined by the drug and tissue properties.

The drug concentration in the blood of skin tissue (TP) depends on the drugs from the tissue of papillary dermis, exchange with the systemic blood (SP) due to blood perfusion, and binding to proteins. Since blood capillaries are distributed within the papillary dermis, drug transport within the capillaries is not considered. Therefore, the drug concentration is a function of time only, as

9 $$\frac{d{C}_{\mathrm{FD},\mathrm{TP}}}{dt}={P}_{\mathrm{FD}}\frac{{S}_{\mathrm{BV}}}{{V}_{\mathrm{TIS}}}\left({C}_{\mathrm{FD},\mathrm{IS}}-{C}_{\mathrm{FD},\mathrm{TP}}\right)+\omega \left({C}_{\mathrm{FD},\mathrm{SP}}-{C}_{\mathrm{FD},\mathrm{TP}}\right)-\frac{d{C}_{\mathrm{BD},\mathrm{TP}}}{dt}$$

in which $\omega$ is the blood perfusion rate. ${C}_{\mathrm{FD},\mathrm{SP}}$ is the concentration of free drugs in the systemic blood. By applying the same assumption of ${K}_{\mathrm{BL}}={C}_{\mathrm{BD},\mathrm{TP}}/{C}_{\mathrm{FD},\mathrm{TP}}$, Eq. (9) can be rewritten as

10 $$\frac{d{C}_{\mathrm{FD},\mathrm{TP}}}{dt}=\frac{{P}_{\mathrm{FD}}}{1+{K}_{\mathrm{BL}}}\frac{{S}_{\mathrm{BV}}}{{V}_{\mathrm{TIS}}}\left({C}_{\mathrm{FD},\mathrm{IS}}-{C}_{\mathrm{FD},\mathrm{TP}}\right)+\frac{\omega }{1+{K}_{\mathrm{BL}}}\left({C}_{\mathrm{FD},\mathrm{SP}}-{C}_{\mathrm{FD},\mathrm{TP}}\right)$$

The concentration of free drugs in the systemic blood is determined by the drug exchange with the tissue blood, plasma clearance, and binding to proteins, as

11 $$\frac{d{C}_{\mathrm{FD},\mathrm{SP}}}{dt}=N\frac{{\int }_{{V}_{\mathrm{PD}}}\omega {C}_{\mathrm{FD},\mathrm{TP}}dv-\omega {\int }_{{V}_{\mathrm{PD}}}{C}_{\mathrm{FD},\mathrm{SP}}dv}{{V}_{\mathrm{SP}}}-{k}_{\mathrm{clc}}{C}_{\mathrm{FD},\mathrm{SP}}-\frac{d{C}_{\mathrm{BD},\mathrm{SP}}}{dt}$$

where $N$ is the number of microneedles on a patch. ${k}_{\mathrm{clc}}$ stands for the plasma clearance rate of the free drug. ${V}_{\mathrm{SP}}$ is the volume of the systemic blood. ${C}_{\mathrm{BD},\mathrm{SP}}$ is the concentration of bound drug in the systemic blood. Based on the same assumption for the process of drug binding to proteins, Eq. (11) can be converted to

12 $$\frac{d{C}_{\mathrm{FD},\mathrm{SP}}}{dt}=N\frac{{\int }_{{V}_{\mathrm{PD}}}\omega {C}_{\mathrm{FD},\mathrm{TP}}dv-\omega {\int }_{{V}_{\mathrm{PD}}}{C}_{\mathrm{FD},\mathrm{SP}}dv}{{V}_{\mathrm{SP}}\left(1+{K}_{\mathrm{BL}}\right)}-{k}_{\mathrm{clc}}\frac{{C}_{\mathrm{FD},\mathrm{SP}}}{1+{K}_{\mathrm{BL}}}$$

### Model Geometry

**Fig. 2:**
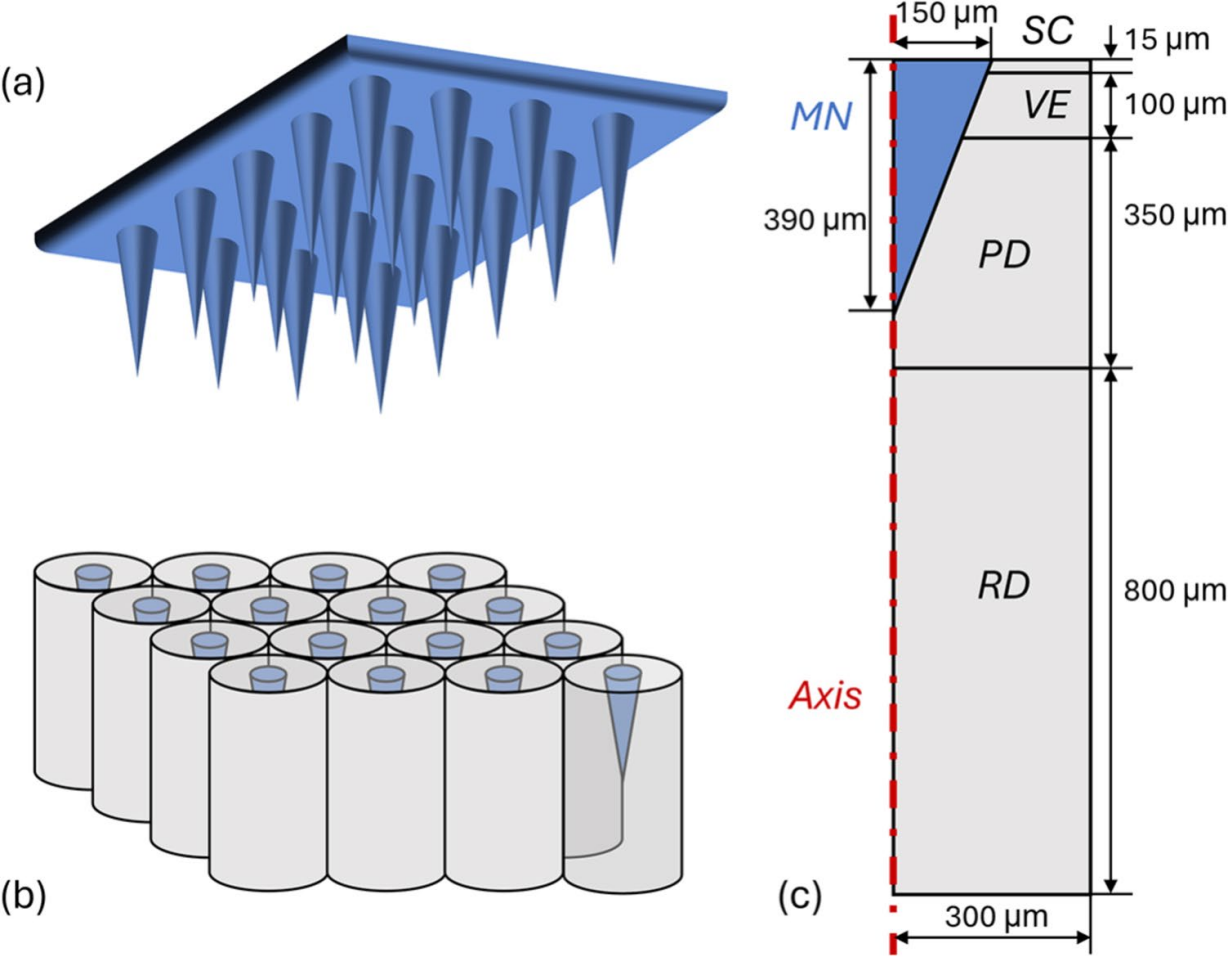
Model geometry. Schematic diagrams showing (**a**) the microneedle array attached to the supporting patch, (**b**) the representative elementary volume of microneedles inserted into the skin for transdermal delivery, and (**c**) the 2-dimensional axisymmetric configuration used for simulations. Since microneedles are typically uniformly distributed on the supporting patch, a single representative elementary volume is used to model the delivery behaviour of an individual microneedle.

A two-dimensional axisymmetric model is used as the representative elementary volume, as microneedles are typically arranged uniformly on the supporting patch, as shown in Fig. 2. In this study, the array consists of 10 × 10 conical microneedles spaced 600 μm apart, each with a height of 390 μm and a base radius of 150 μm. The thickness of the stratum corneum is 15 μm, while the viable epidermis and papillary dermis layers are 100 μm and 350 μm thick [27]. The microneedles can fully penetrate the top layers to reach the papillary dermis. The thickness of the reticular dermis layer is 800 μm [27], representing approximately half the distance between the papillary and reticular plexuses. The computational domain is discretised with approximately 60,000 triangular elements based on a mesh sensitivity analysis, with the finest elements of 0.005 μm concentrated near the microneedle–tissue interface for improved accuracy.

### Model Parameters

The tissue and drug properties are assumed to remain constant throughout the simulated drug delivery period, as the duration considered is shorter than the characteristic timescale of tissue remodelling and growth. The values of the tissue properties and the transport-related parameters of the ten representative drugs are given in Tables I and II, respectively. The drugs include fluorouracil (5-FU), temozolomide (TMZ), carmustine (BCNU), cisplatin (CDDP), dexamethasone (DEX), methotrexate (MTX), doxorubicin (DOX), paclitaxel (PTX), insulin (INS), and immunoglobulin G (IgG).

**Table I:** Model Parameter for Tissue Properties

Parameter (unit)	VE	PD	RD	Source
${\kappa }_{\mathrm{tis}}\left({\mathrm{m}}^{2}\right)$	$1.0\times {10}^{-16}$	$1.0\times {10}^{-16}$	$1.0\times {10}^{-16}$	[30]
${\mu }_{\mathrm{is}}\left(\mathrm{Pa}\cdot \mathrm{s}\right)$	$7.8\times {10}^{-4}$	$7.8\times {10}^{-4}$	$7.8\times {10}^{-4}$	[31]
${\rho }_{\mathrm{is}}\left(\mathrm{kg}/{\mathrm{m}}^{3}\right)$	$1000$	$1000$	$1000$	[32]
${\pi }_{\mathrm{bl}}\left(\mathrm{Pa}\right)$	$-$	$2670$	$-$	[33]
${\pi }_{\mathrm{is}}\left(\mathrm{Pa}\right)$	$-$	$1330$	$-$	[33]
${\sigma }_{\mathrm{T}}\left(1\right)$	$-$	$0.91$	$-$	[33]
${L}_{\mathrm{bl}}\left(\mathrm{m}/\mathrm{Pa}/\mathrm{s}\right)$	$-$	$2.7\times {10}^{-12}$	$-$	[33]
${p}_{\mathrm{bl}}\left(\mathrm{Pa}\right)$	$-$	$2080$	$-$	[33]
${S}_{\mathrm{bl}}/{V}_{\mathrm{tis}}\left({\mathrm{m}}^{-1}\right)$	$-$	$6.0\times {10}^{3}$	$-$	[34]
${{L}_{\mathrm{ly}}S}_{\mathrm{ly}}/{V}_{\mathrm{tis}}\left(1/\mathrm{Pa}/\mathrm{s}\right)$	$-$	$4.2\times {10}^{-7}$	$-$	[33]
${p}_{\mathrm{ly}}\left(\mathrm{Pa}\right)$	$-$	$0$	$-$	[33]
$\omega\left({\mathrm{s}}^{-1}\right)$	$-$	$8.5\times {10}^{-4}$	$-$	[35]
${V}_{\mathrm{SP}} (\mathrm{L})$	$-$	$5.0$	$-$	[36]

**Table II:** Model parameters of therapeutic agents

Parameter (unit)	5-FU	TMZ	BCNU	CDDP	DEX	MTX	DOX	PTX	INS	IgG	Baseline^*^
$MW\;\left(Da\right)$	$130.08$ [29]	$194.15$ [37]	$214.05$ [38]	$300.01$ [29]	$392.47$ [39]	$454.44$ [40]	$543.52$ [41]	$853.91$ [42]	$5808$ [43]	$146000$ [29]	$-$
$P_{CI-IS\;}\left(1\right)$	$1.0$ [26]	$1.0$ [26]	$1.0$ [26]	$1.0$ [26]	$1.0$ [26]	$1.0$ [26]	$1.0$ [26]	$1.0$ [26]	$1.0$ [26]	$1.0$ [26]	$1.0$
$P_{CM-IS}\;\left(1\right)$	$0.1$ [29]	$0.015$ [44]	$10.3$ [45]	$0.006$ [46]	$95.50$ [29]	$0.01$ [29]	$0.3$ [47]	$3162.3$ [48]	$0$ [29]	$0$ [29]	$1.0$
$K\;\left(1\right)$	$0.1$ [49]	$0.18$ [50]	$5.0$ [45]	$4.4$ [51]	$2.3$ [52]	$0.7$ [53]	$3.0$ [54]	$5.1$ [55]	$0$ [29]	$0$ [29]	$1.0$
$D_{FD}\;\left(\times10^{-9}m^2/s\right)$	$0.46$ [28]	$0.34$ [28]	$0.32$ [28]	$0.25$ [28]	$0.20$ [28]	$0.18$ [28]	$0.16$ [28]	$0.11$ [28]	$0.03$ [28]	$2.0\times {10}^{-5}$ [29]	$0.01$
$P_{FD}\;\left(\times10^{-6}m/s\right)$	$3.0$ [29]	$0.04$ [56]	$2.33$ [45]	$0.03$ [29]	$48.61$ [29]	$0.05$ [29]	$0$ [57]	$0.02$ [48]	$1.85\times {10}^{-7}$ [29]	$0$ [29]	$0.01$
${k}_{\mathrm{ra}}$($\times {{10}^{-3}\mathrm{s}}^{-1}$)	$0.56$ [29]	$0.11$ [58]	$0.11$ [45]	$0.08$ [29]	$0.11$ [29]	$0.15$ [29]	$0.58$ [31]	$6.8\times {10}^{-4}$ [48]	$0.048$ [59]	$2.8\times {10}^{-4}$ [29]	$0.1$
${k}_{\mathrm{clc}}$(${\times {10}^{-3}\mathrm{s}}^{-1}$)	$1.16$ [60]	$0.11$ [56]	0.52 [61]	$0.50$ [62]	$0.048$ [63]	$0.055$ [64]	$2.43$ [65]	$0.11$ [66]	$2.22$ [59]	$3.1\times {10}^{-4}$ [67]	$0.1$

*Baseline values were selected according to the parameter ranges of the ten drugs

Diffusivity in the tissue quantifies the ability of a drug to migrate within the tissue interstitial space via thermal motion. The diffusivity (in ${\mathrm{m}}^{2}/\mathrm{s}$) of small-molecule drugs with molecular weights ($MW$, in Da) ranging from 32 to 69,000 is given by [28]

13 $${D}_{\mathrm{F},\mathrm{IS}}=1.778\times {10}^{-8}{\left(MW\right)}^{-0.75}, 32<MW<69000$$

whereas the diffusivity of IgG, whose molecular weight exceeds this range, is obtained from the literature [29]. In the absence of reported transvascular permeability data for individual drugs in skin tissues, the corresponding values determined in the brain are adopted, considering the fact that the capillaries in both tissues are continuous and lack fenestrations. The parametric analysis is designed to examine the influence of each key drug property, with the parameter ranges selected to be sufficiently broad to encompass, and slightly extend beyond, the values reported for the ten representative drugs; they are specified in the following section, where the impact of each parameter is discussed.

### Boundary Conditions

The trans-epidermal water loss represents the diffusion-driven escape of water vapour through the stratum corneum into the atmosphere, depending on the environment. Based on mass conservation, the evaporative flux at the surface of the stratum corneum is dynamically balanced with the water flux at the stratum corneum-viable epidermis interface, which is set to $3.43\times {10}^{-9} \mathrm{m}/\mathrm{s}$ under 0.1 m/s air velocity and conditions of 80% relative humidity [68]. The drug flux at this interface is assumed to be zero, as most drugs are unable to penetrate the stratum corneum. Similarly, the drug flux at the bottom surface of the microneedle is zero, assuming complete encapsulation of the drug within the microneedle.

The drug transfer between the microneedle and the adjacent skin tissue is described by

14 $$\left\{\begin{array}{l}{C}_{\mathrm{FD},\mathrm{tis}}={K}_{\mathrm{MT}}{C}_{\mathrm{FD},\mathrm{MN}}\\ -{D}_{\mathrm{FD},\mathrm{tis}}\frac{\partial {C}_{\mathrm{FD},\mathrm{tis}}}{\partial n}={-D}_{\mathrm{FD},\mathrm{MN}}\frac{\partial {C}_{\mathrm{FD},\mathrm{MN}}}{\partial n}\end{array}\right.$$

where the parameter ${K}_{\mathrm{MT}}$ is the drug partition coefficient between the skin tissue and the microneedle. The microneedle surface is assumed to be rigid and imposes a no-slip condition on interstitial fluid flow. Continuous boundary conditions are applied at the interfaces between adjacent skin layers [30, 69], while a symmetry boundary is imposed at the lateral edge of the computational domain. Zero-flux conditions for both fluid and drug are applied at the bottom boundary [27].

### Numerical Methods

The governing equations are solved in COMSOL Multiphysics to obtain the numerical solutions, subject to the parameters and boundary conditions. The interstitial fluid transport model is first solved under quasi-steady-state conditions to establish the fluid flow field, which is subsequently imported into the drug transport model to predict the drug’s temporal and spatial profiles in the microneedle, skin layers, tissue blood, and systemic circulation. At the beginning of the simulation, all drugs are assumed to be fully loaded within the microneedle, while their concentrations in the skin tissues and circulatory systems are set to zero.

### Qualification of Delivery Outcomes

The performance of microneedle-mediated transdermal delivery under different conditions is evaluated according to the following qualification indices.

#### Drug Accumulation

The drug accumulation in each skin layer is assessed using the normalised spatially averaged concentration (${C}_{\mathrm{nor},\mathrm{avg}}$) in the particular tissue compartment, defined as

15 $${C}_{\mathrm{nor},\mathrm{avg}}=\frac{\sum {C}_{\mathrm{i}}{V}_{\mathrm{i}}}{{C}_{\mathrm{MN},0}\sum {V}_{\mathrm{i}}}=\frac{\sum {C}_{\mathrm{i}}{V}_{\mathrm{i}}}{{C}_{\mathrm{MN},0}{V}_{\mathrm{TIS}}}$$

in which ${C}_{\mathrm{i}}$ is the local concentration of free drugs, and ${V}_{\mathrm{i}}$ is the tissue volume. ${C}_{\mathrm{MN},0}$ is the initial drug concentration in the microneedle.

#### Drug Exposure

The treatment efficiency can be associated with the drug exposure, defined as the integration of normalised spatially averaged concentration over time (*T*), as

16 $${AUC}_{\mathrm{nor},\mathrm{T}}={\int }_{0}^{T}{C}_{\mathrm{nor},\mathrm{avg}}dt$$

## Results

### Baseline Study

**Fig. 3:**
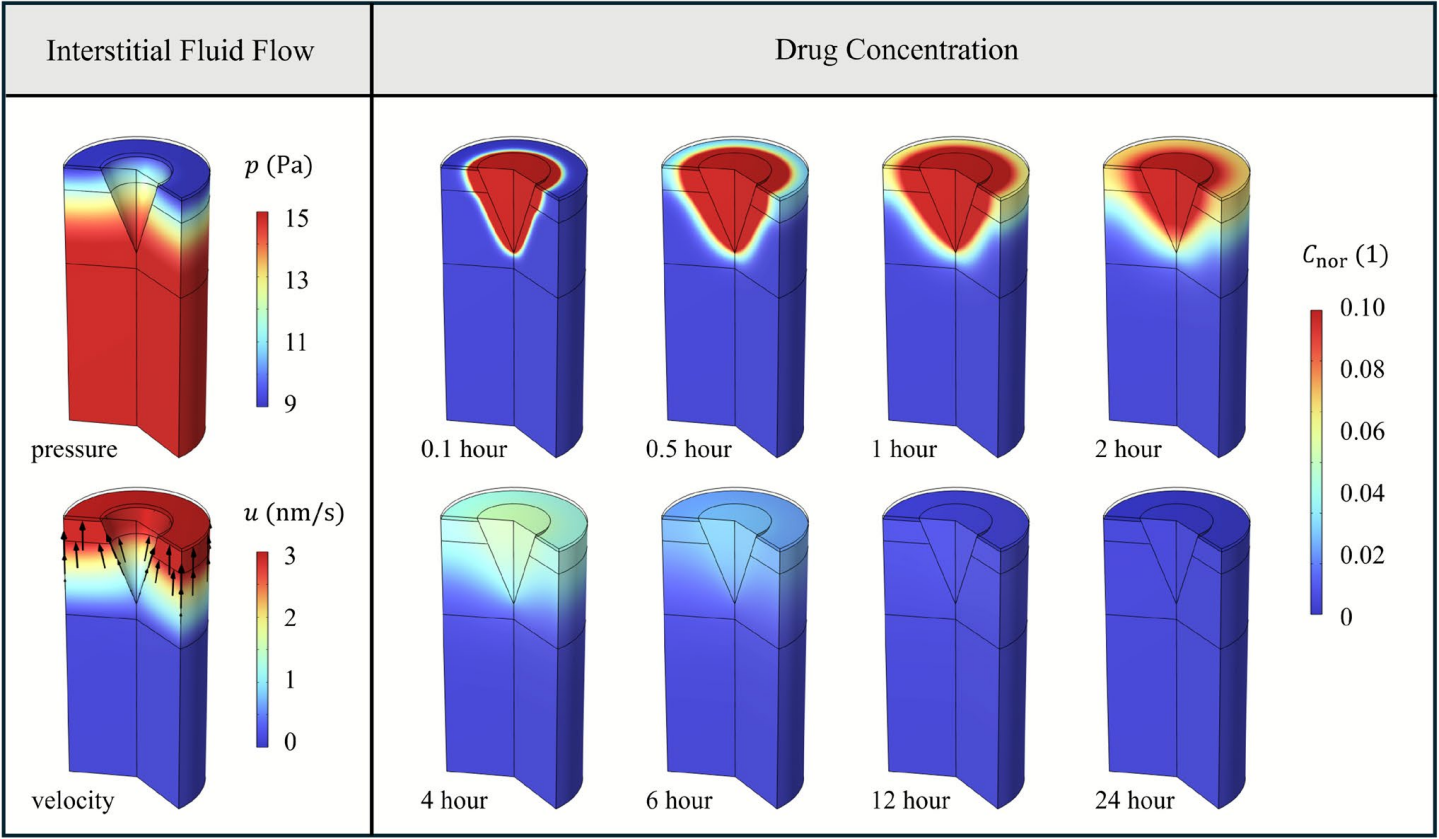
Interstitial fluid flow and drug concentration distribution. The black arrows in the velocity contour stand for the flow directions.

The drugs released from the microneedles transfer into the interstitial spaces of the skin tissues through coupled diffusion and convection mechanisms, with the former governed by concentration gradients and the latter driven by interstitial fluid flow. The fluid transport model is solved within the viable skin layers to predict the interstitial fluid flow. As illustrated in Fig. 3, the pressure within the deeper tissues is higher and gradually decreases towards the skin surface, induced by the trans-epidermal water loss. This pressure gradient results in the upward movement of interstitial fluid towards the surface. The most prominent flow occurs at the stratum corneum-viable epidermis interface and progressively diminishes with depth into the dermal region.

The contours of drug concentration in Fig. 3 indicate that the microneedle effectively delivers the drug into the skin tissue. Initially, the drug diffuses into the tissues adjacent to the microneedle insertion sites and subsequently disperses into deeper layers over time. Concurrently, the drug concentration within the microneedle declines as release continues. Eventually, the concentration decreases to a relatively low level and reaches a near-uniform distribution across both the microneedle and the skin tissues.

**Fig. 4:**
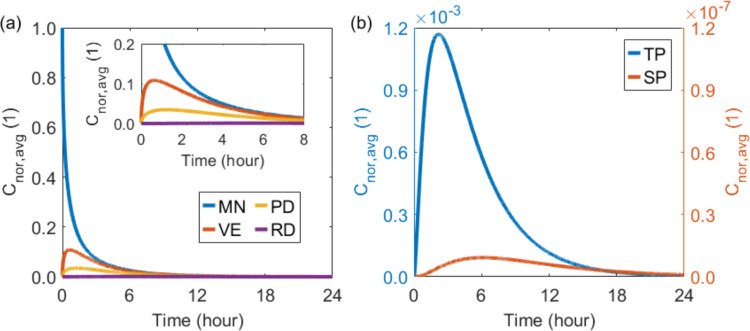
Drug concentration–time profiles in the microneedle and skin layers (**a**), and in the plasma (**b**).

The temporal profiles of drug concentration are compared across the microneedle, various skin layers, and blood compartments in Fig. 4. The modelling results show that the drug concentration within the microneedle continuously decreases over time owing to the release dynamics. Driven by the concentration gradient, the drug concentration in the skin tissues initially rises to a peak and subsequently declines as the supply from the microneedle diminishes. Drug accumulation is most pronounced in the viable epidermis, followed by the papillary dermis and reticular dermis in order. Moreover, the drug concentrations in both the tissue blood and systemic blood exhibit similar temporal trends, characterised by an initial increase to a peak followed by a gradual decrease. However, the concentration in the systemic blood is several orders of magnitude lower than that in the tissue blood. This is because the drug must traverse the tissue blood to enter the systemic circulation, which has a significantly larger volume, and the drugs in the systemic blood are continuously removed through plasma clearance processes.

### Effect of Drug Diffusivity in the Microneedle

**Fig. 5:**
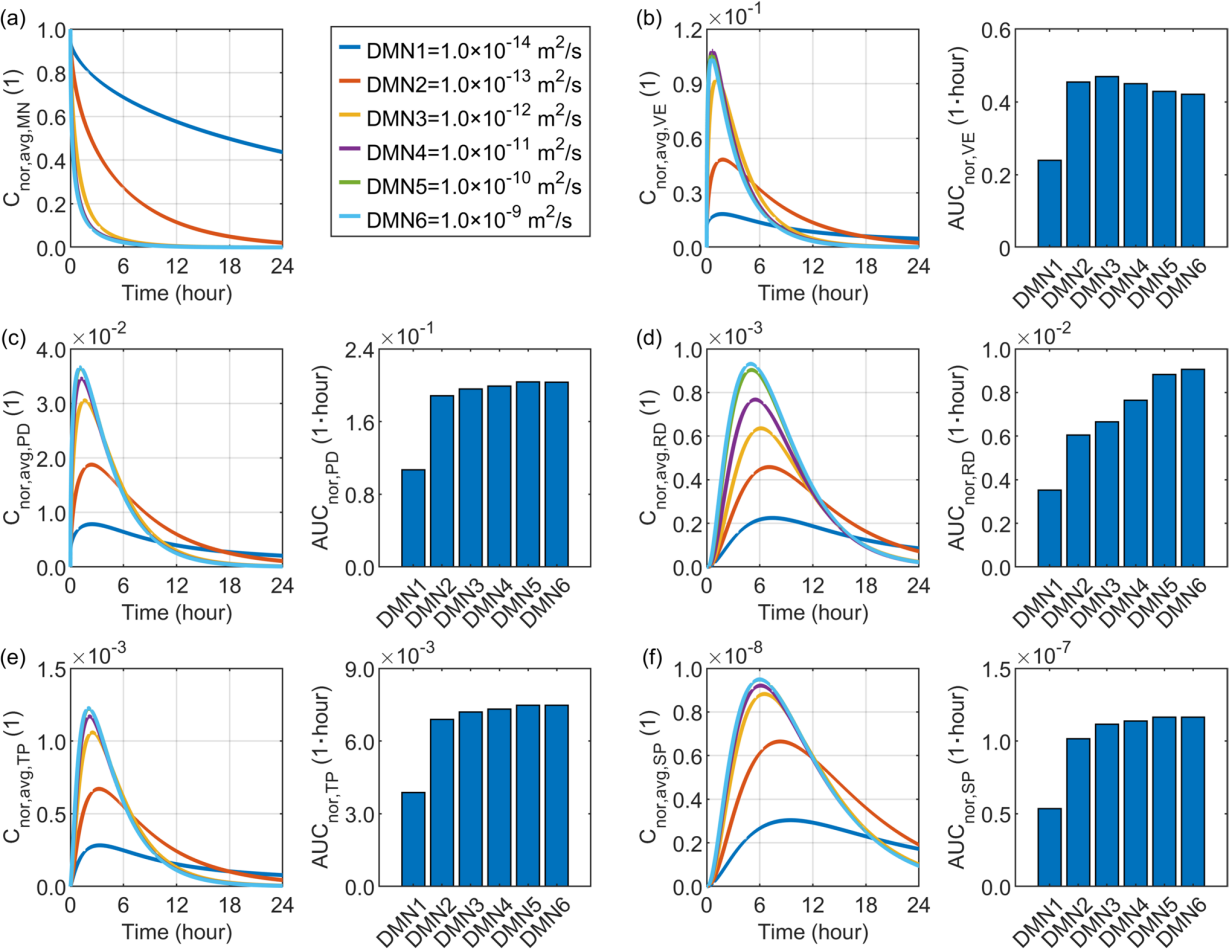
Delivery outcomes for drugs with different diffusivities in the microneedle. Subplot (**a**) shows the model-predicted drug concentration in the microneedle, while subplots (**b**) to (**f**) present drug concentration and exposure in the viable epidermis, papillary dermis, reticular dermis, tissue blood, and systemic blood, respectively.

The range of $1.0\times {10}^{-14} {\mathrm{m}}^{2}/\mathrm{s}$ to $1.0\times {10}^{-9} {\mathrm{m}}^{2}/\mathrm{s}$ is selected to examine the effect of drug diffusivity in the microneedle (${D}_{\mathrm{MN}}$​) on drug delivery outcomes in the skin and the blood circulatory system, as shown in Fig. 5. As ${D}_{\mathrm{MN}}$​ increases, drug release from the microneedle is effectively accelerated due to the enhanced molecular mobility. This rapid and unsustainable drug supply leads to a higher peak concentration but a faster decline in the papillary dermis, reticular dermis, tissue blood, and systemic blood. In contrast, the viable epidermis exhibits its highest peak concentration when ${D}_{\mathrm{MN}}$​ is $1.0\times {10}^{-11} {\mathrm{m}}^{2}/\mathrm{s}$, whereas further increases in this diffusivity slightly reduce drug accumulation in this layer. Notably, rather than the peak concentration, drug exposure is governed by the time course of drug concentration, as shown in Eq. (16). Results demonstrate that raising ${D}_{\mathrm{MN}}$ to $1.0\times {10}^{-12} {\mathrm{m}}^{2}/\mathrm{s}$ markedly enhances drug exposure in the viable epidermis, whereas further increases cause a gradual decline. For the remaining skin layers and blood compartments, drug exposure increases monotonically with the increase in this parameter. The rate of increase in the papillary dermis, tissue blood, and systemic blood slows once ${D}_{\mathrm{MN}}$​ exceeds $1.0\times {10}^{-13} {\mathrm{m}}^{2}/\mathrm{s}$, while the reticular dermis continues to show a relatively rapid rise, as it is the deepest layer to receive the drug.

### Effect of the Tissue-Microneedle Drug Partition Coefficient

This coefficient is commonly set to $1.0$, as polymeric or hydrogel-based microneedles can be regarded as aqueous phases similar to skin tissue. It is worth noting that its value may vary depending on several factors, including the microneedle formulation and material composition, the drug’s hydrophobicity or hydrophilicity, and its surface charge. An arbitrary range from $0$ to $100$ is selected to examine its influence, where a value of 0 represents an extreme case in which the drug is completely insoluble in the tissue.

**Fig. 6:**
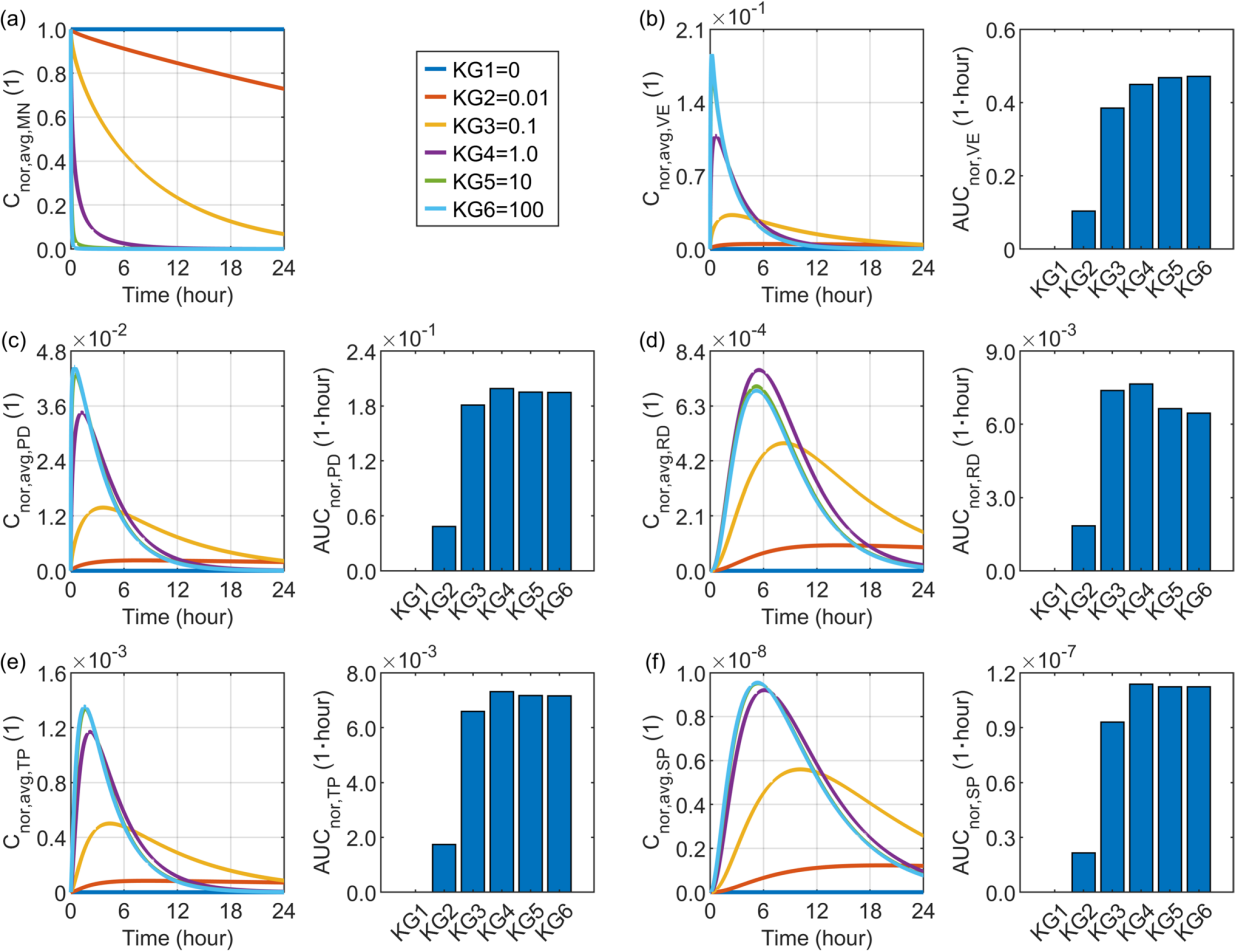
Delivery outcomes for drugs with different tissue-microneedle partition coefficients. Subplot (**a**) shows the model-predicted drug concentration in the microneedle, while subplots (**b**) to (**f**) present drug concentration and exposure in the viable epidermis, papillary dermis, reticular dermis, tissue blood, and systemic blood, respectively.

Figure 6 compares the delivery outcomes under different tissue-microneedle partition coefficients. A higher partition coefficient greatly shortens the release period of the loaded drug from the microneedle. This shift in the release profile allows the drug concentration in the viable epidermis to reach a higher peak, followed by a faster decline over time. However, the overall drug exposure during the 24-h release window shows a monotonic increase with this partition coefficient. In contrast, although increasing this coefficient effectively raises the peak concentrations in the papillary dermis and blood compartments, further increasing it beyond $1.0$ causes the concentration to drop to a lower level earlier. Consequently, the 24-h drug exposure exhibits a sharp rise as the partition coefficient increases from $0$ to $1.0$, while additional increases lead to a reduction in local drug exposure. This trend is particularly evident in the reticular dermis, where the concentration reaches its maximum peak at a partition coefficient of 1.0. Moreover, at this value, the concentration is maintained at a higher level over time compared with cases in which the partition coefficient exceeds 1.0.

### Effect of Drug Diffusivity in the Skin Tissue

**Fig. 7:**
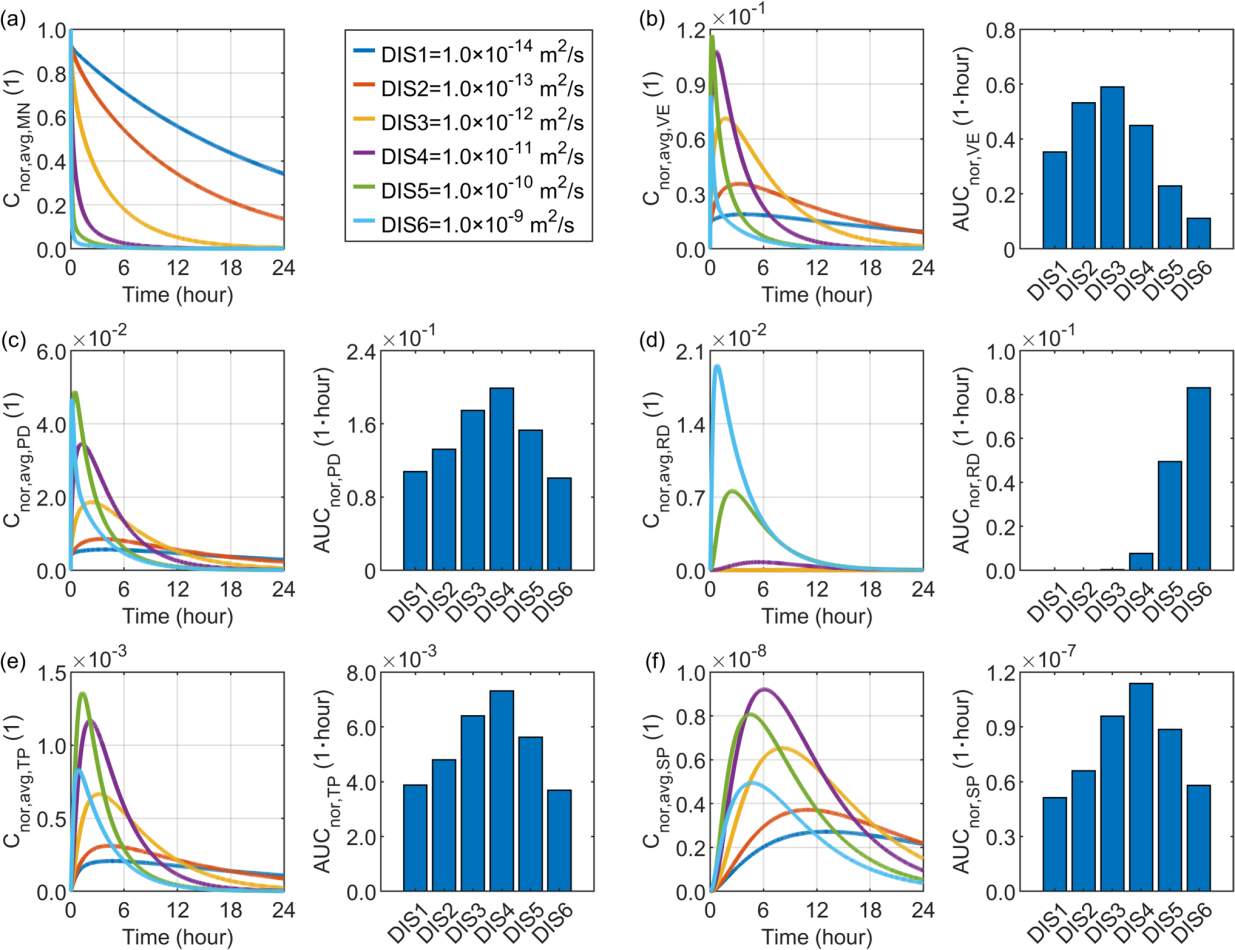
Delivery outcomes for drugs with different diffusivities in skin tissues. Subplot (**a**) shows the model-predicted drug concentration in the microneedle, while subplots (**b**) to (**f**) present drug concentration and exposure in the viable epidermis, papillary dermis, reticular dermis, tissue blood, and systemic blood, respectively.

Drug diffusivity in the skin tissue reflects the ability of drug molecules to move within the tissue due to their thermal motion. Its impact on delivery outcomes is examined by varying this factor from $1.0\times {10}^{-14} {\mathrm{m}}^{2}/\mathrm{s}$ to $1.0\times {10}^{-9} {\mathrm{m}}^{2}/\mathrm{s}$. The comparisons in Fig. 7 show that higher diffusivity facilitates more rapid drug release from the microneedle, as it enhances the concentration gradient across the microneedle-tissue interface. Although this accelerated release allows more drug to rapidly enter the skin after administration, high diffusivity can also promote drug transfer to downstream layers. Consequently, the drug concentration exhibits a stronger response to increases in diffusivity.

The most effective drug accumulation occurs when these two opposing mechanisms reach a balance in each compartment. Simulation results indicate that, although a diffusivity of $1.0\times {10}^{-10} {\mathrm{m}}^{2}/\mathrm{s}$ produces the highest peak concentration in the viable epidermis, the optimal drug exposure in this layer is achieved at $1.0\times {10}^{-12} {\mathrm{m}}^{2}/\mathrm{s}$, since the drug exposure is subject to concentration time course. In the papillary dermis, the highest drug exposure occurs at $1.0\times {10}^{-11} {\mathrm{m}}^{2}/\mathrm{s}$, while the peak concentration is maximal at $1.0\times {10}^{-10} {\mathrm{m}}^{2}/\mathrm{s}$. For tissue blood and systemic blood, the peak concentrations are reached at $1.0\times {10}^{-10} {\mathrm{m}}^{2}/\mathrm{s}$ and $1.0\times {10}^{-11} {\mathrm{m}}^{2}/\mathrm{s}$, respectively, whereas the most effective drug exposure in both compartments occurs at $1.0\times {10}^{-11} {\mathrm{m}}^{2}/\mathrm{s}$. In contrast, drug exposure in the reticular dermis increases steadily with this diffusivity, as this deepest skin layer accumulates more drug when this factor is higher.

### Effect of Drug Elimination Rate

**Fig. 8:**
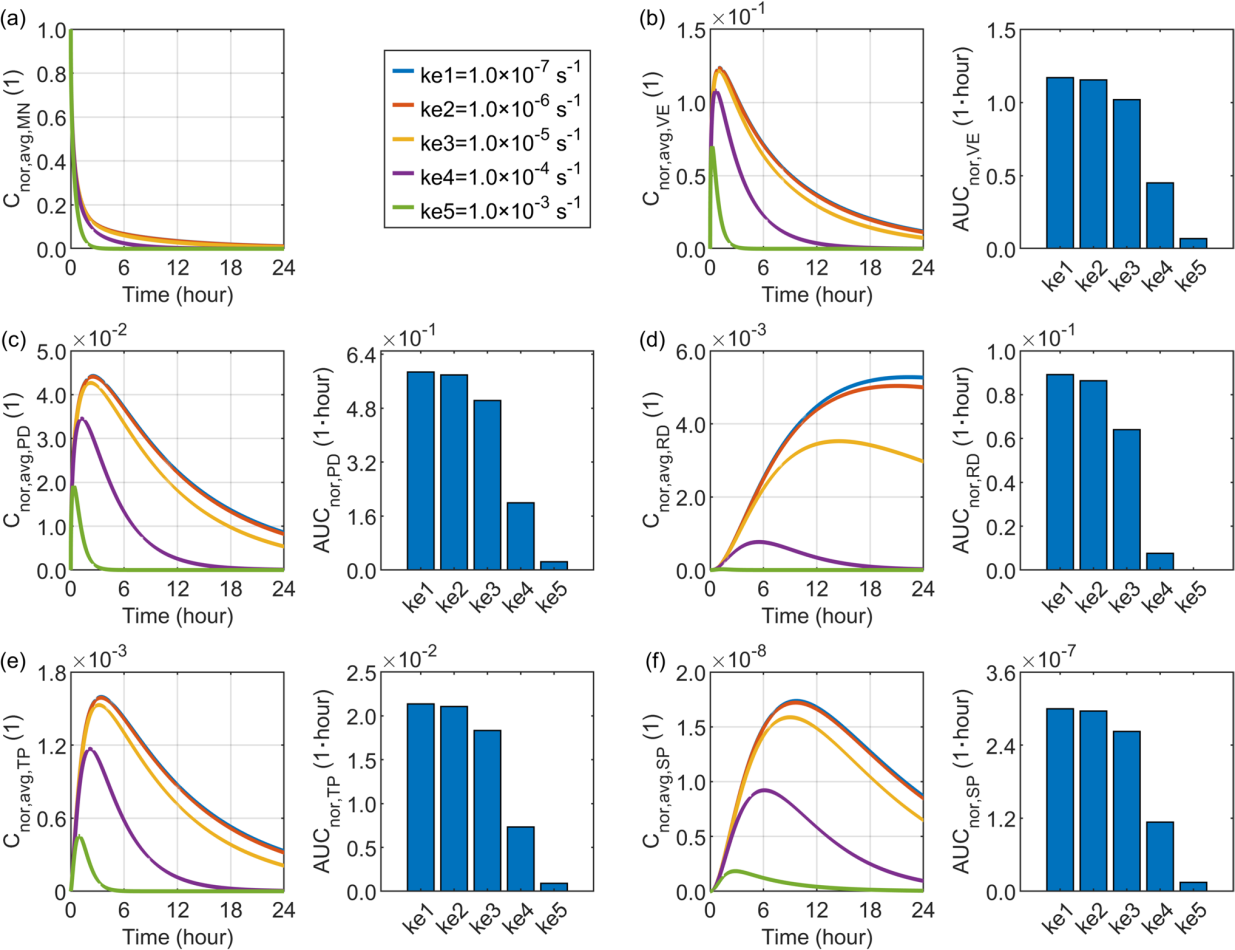
Delivery outcomes for drugs with different elimination rates in skin tissues. Subplot (**a**) shows the model-predicted drug concentration in the microneedle, while subplots (**b**) to (**f**) present drug concentration and exposure in the viable epidermis, papillary dermis, reticular dermis, tissue blood, and systemic blood, respectively.

The elimination rate represents the timescale of drug reactions with biomolecules and the physical degradation processes that convert the drug into other substances. This factor varies considerably among different drugs. A range from $1.0\times {10}^{-7} {\mathrm{s}}^{-1}$ to $1.0\times {10}^{-3} {\mathrm{s}}^{-1}$ is selected to cover the values listed in Table II. Figure 8 compares the delivery outcomes for drugs with different elimination rates. As expected, drugs with higher elimination rates exhibit lower concentrations in all skin layers, as they are more rapidly depleted. Consequently, the concentration gradient across the microneedle-tissue interface increases, thereby accelerating drug release from the microneedle. Moreover, the drug concentration and exposure in the tissue blood and systemic blood exhibit a similar pattern to those observed in the papillary dermis, as blood capillaries are embedded in this layer, and the systemic blood is downstream of the tissue blood in drug transport.

### Effect of Drug–protein Binding Coefficient

The drug–protein binding coefficient is defined as the ratio between the concentration of bound drug and that of the drug in its free form. Although the binding process occurs much more rapidly than the overall drug delivery process, this coefficient varies considerably among different drugs. Based on the values summarised in Table II, a range of $0-100$ is adopted in this study to evaluate its influence.

**Fig. 9:**
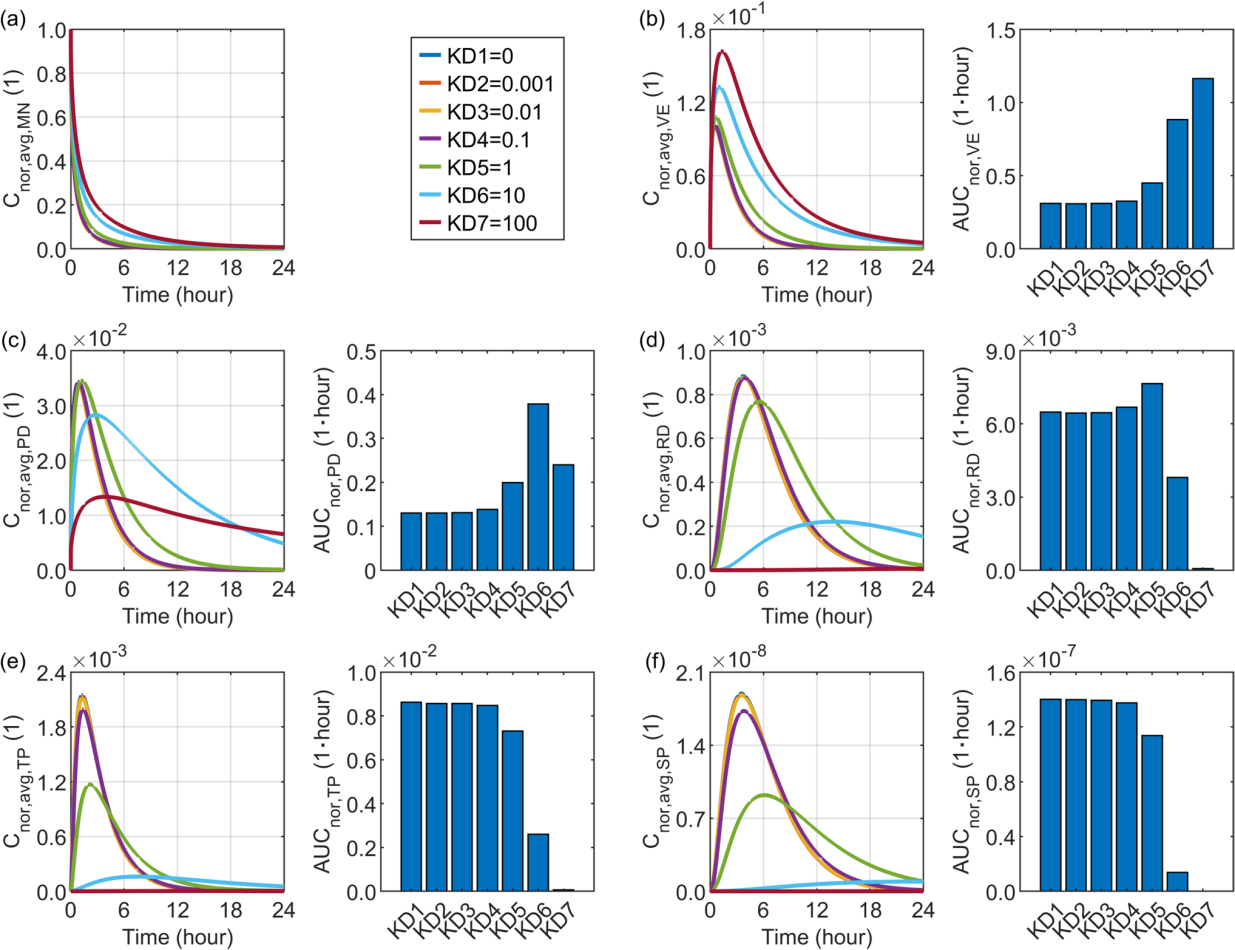
Delivery outcomes for drugs with different protein binding coefficients. Subplot (**a**) shows the model-predicted drug concentration in the microneedle, while subplots (**b**) to (**f**) present drug concentration and exposure in the viable epidermis, papillary dermis, reticular dermis, tissue blood, and systemic blood, respectively.

Figure 9 presents the delivery outcomes for drugs with varying protein-binding coefficients. The results indicate that increasing this parameter greatly slows the temporal evolution of drug concentration within the papillary dermis, where this biochemical interaction takes place. Notably, this delay becomes more pronounced when the coefficient exceeds $1.0$. Such retardation in drug accumulation within the papillary dermis leads to slower drug release from the microneedle and enhanced drug retention in the upper layer of viable epidermis, while drug accumulation in the downstream reticular dermis is likewise delayed. According to the temporal profiles of drug concentration across skin layers, higher values of this coefficient enhance drug exposure in the viable epidermis, whereas optimal exposure is achieved in the papillary and reticular dermis when the coefficient reaches approximately 10 and 1, respectively. Furthermore, it is important to note that drug-protein binding also occurs in the blood plasma. A higher binding coefficient indicates a lower proportion of the drug remaining in its free form. Consequently, drug exposure in both tissue blood and systemic blood decreases as the binding coefficient increases.

### Effect of the Cell Membrane–interstitial Space Partition Coefficient

The drug partition coefficient between the cell membrane and interstitial space is closely governed by the drug’s hydrophobicity or hydrophilicity, as the cell membrane is composed of a lipid bilayer, whereas the interstitial space represents an aqueous environment. This parameter varies markedly among drugs, depending on their molecular structure, and is commonly approximated by the octanol–water partition coefficient. To encompass the potential range of values this factor may assume, as summarised in Table II, a wide range from $0$ to $10000$ is adopted in the present study to examine its influence on drug delivery outcomes.

**Fig. 10:**
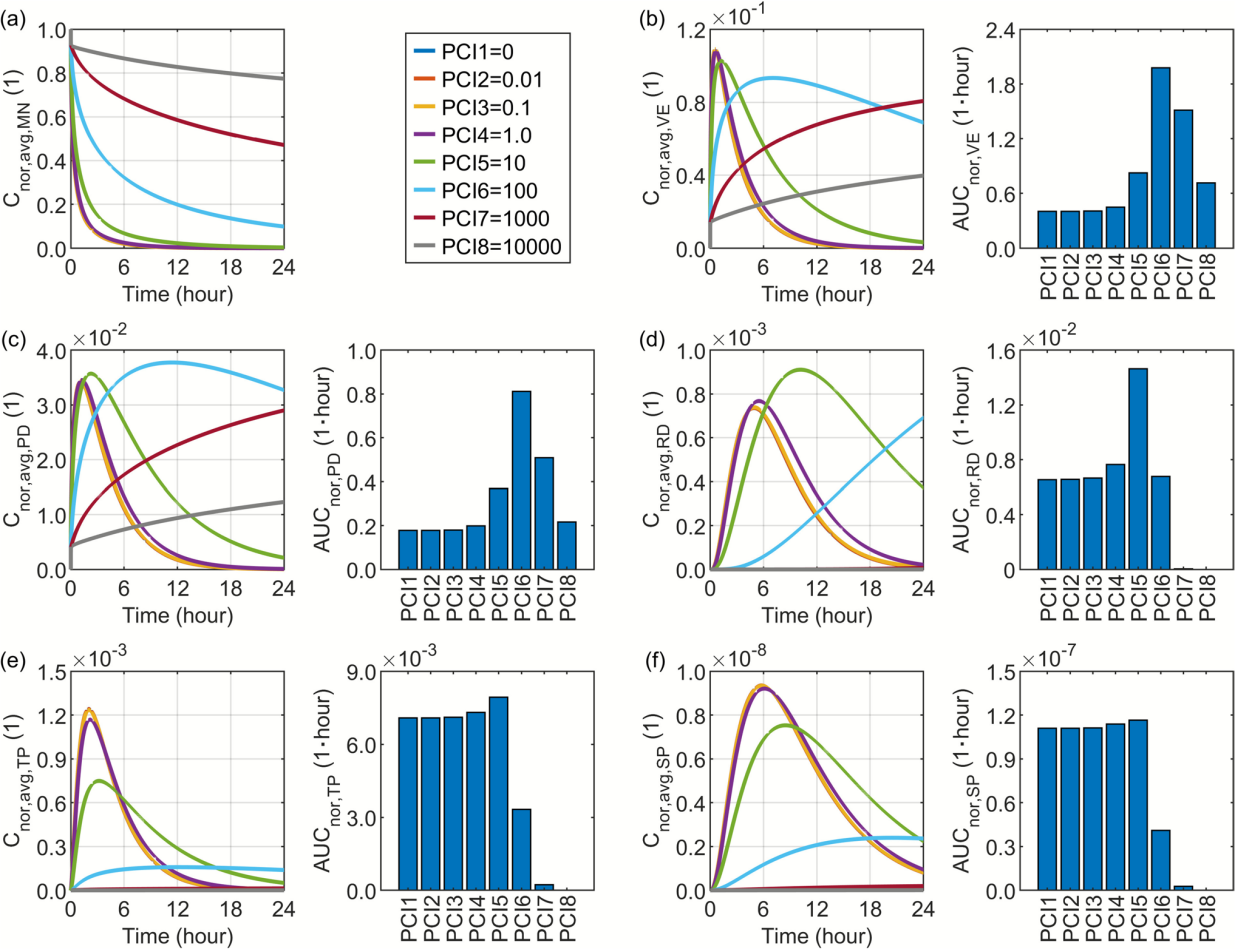
Delivery outcomes for drugs with different partition coefficients between the cell membrane and the interstitial space. Subplot (**a**) shows the model-predicted drug concentration in the microneedle, while subplots (**b**) to (**f**) present drug concentration and exposure in the viable epidermis, papillary dermis, reticular dermis, tissue blood, and systemic blood, respectively.

Figure 10 illustrates the simulated results for drugs with different cell membrane-interstitial space partition coefficients. The modelling predictions indicate that increasing this parameter substantially slows the temporal variation of drug concentration across all skin layers. This phenomenon arises because a larger proportion of the drug partitions into the cell membrane, thereby reducing the proportion available to penetrate deeper tissues or undergo elimination. The effect is relatively minor when the partition coefficient increases from $0$ to $1$, but becomes pronounced at higher values, leading to a significant delay in concentration dynamics. Such delayed responses within the skin layers further influence the drug release from the microneedle, resulting in a more sustained drug supply under conditions of high partition coefficients. A similar delay is also observed in the two blood compartments, as the drugs present in the bloodstream originate from the papillary dermis.

It is noteworthy that drug exposure depends on the temporal evolution of concentration. Therefore, the optimal drug exposure varies across compartments within the 24-h period considered in this study. Specifically, the most substantial drug exposure in the viable epidermis and papillary dermis is achieved at a partition coefficient of $100$, whereas a value of $10$ yields the most effective exposure in the reticular dermis. In contrast, when this coefficient ranges from $0$ to $10$, comparable levels of drug exposure are observed in both the tissue blood and systemic blood, although a slightly higher exposure is noted at a value of 10. However, further increasing this parameter leads to dramatic drops in drug exposure over the examined delivery period.

### Effect of the Cell Interior–interstitial Space Partition Coefficient

The partition coefficient of a drug between the cell interior and interstitial space is commonly assumed to be 1.0 in modelling studies, as both regions are primarily aqueous phases. However, differences in their biochemical compositions and drug transport processes, particularly the internalisation mechanism, may influence the actual drug partitioning between these compartments. In the absence of experimentally reported values, a broad range from $0.01$ to $100$ is arbitrarily selected to investigate its impact.

**Fig. 11:**
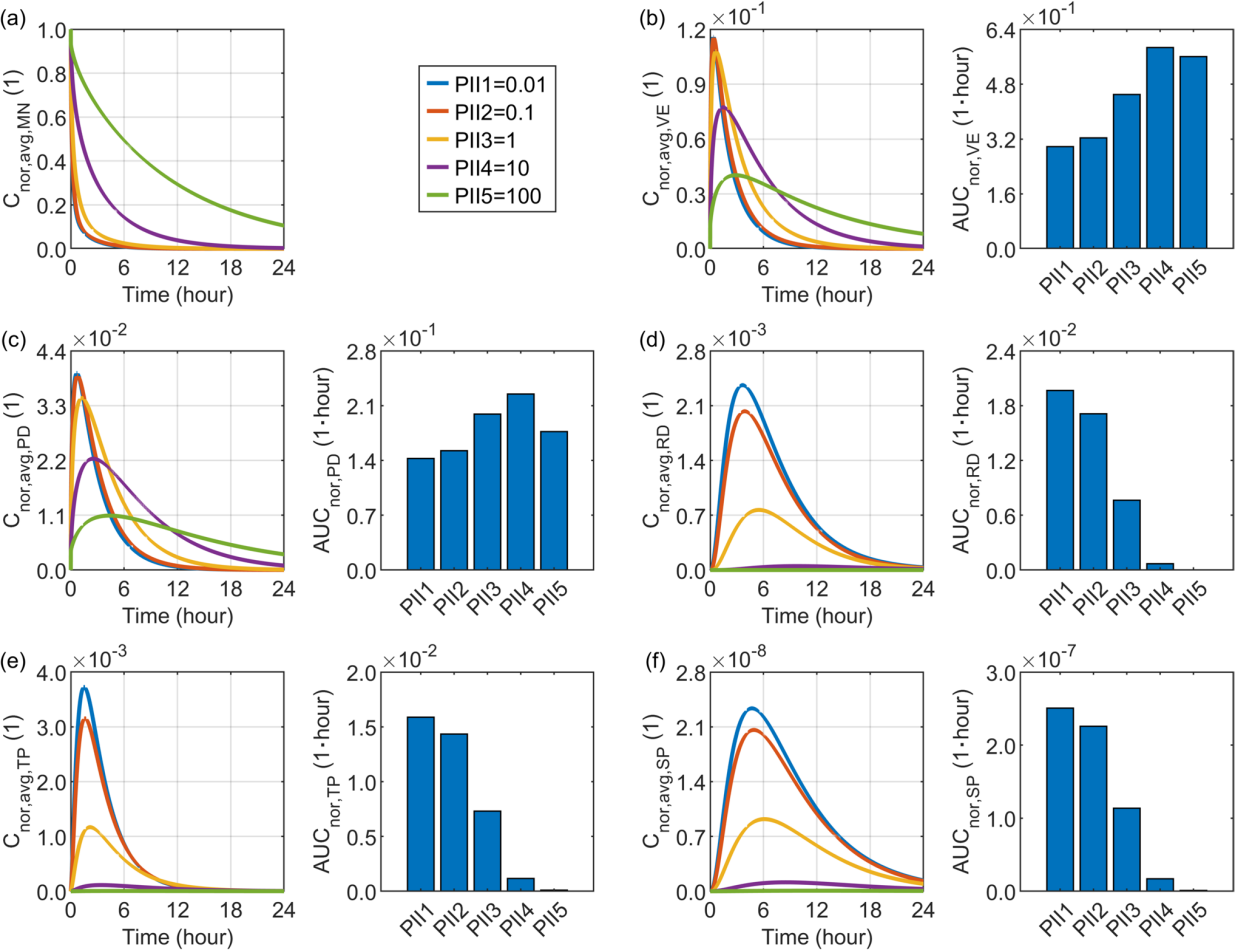
Delivery outcomes for drugs with different partition coefficients between the cell interior and the interstitial space. Subplot (**a**) shows the model-predicted drug concentration in the microneedle, while subplots (**b**) to (**f**) present drug concentration and exposure in the viable epidermis, papillary dermis, reticular dermis, tissue blood, and systemic blood, respectively.

The delivery outcomes for drugs with varying cell interior–interstitial space partition coefficients are presented in Fig. 11. The results demonstrate that in the viable epidermis and papillary dermis, which are both adjacent to the microneedle, increasing this coefficient reduces the maximum achievable concentration but promotes a more gradual and sustained accumulation of the drug over time. Consequently, optimal drug exposure is observed when this parameter is approximately $10$. Moreover, such a gradual temporal concentration profile in the tissues surrounding the microneedle not only slows the release of the drug from the microneedle but also results in lower concentrations in downstream compartments, including the reticular dermis, tissue blood, and systemic circulation. Therefore, the most effective drug exposure in these compartments occurs when the drug exhibits a low propensity to accumulate within cells, which in turn facilitates deeper penetration into the skin tissue and bloodstream.

### Effect of Drug Transvascular Permeability

Transvascular permeability represents the ability of drug molecules to penetrate the capillary wall. Together with the capillary density in the papillary dermis, this parameter directly determines drug availability in the plasma compartments and other tissues, except for the local skin region where the microneedles are applied. A broad range from 0 to $1.0\times {10}^{-4} \mathrm{m}/\mathrm{s}$ is selected to evaluate its impact, which sufficiently covers the parameter values listed in Table II.

**Fig. 12:**
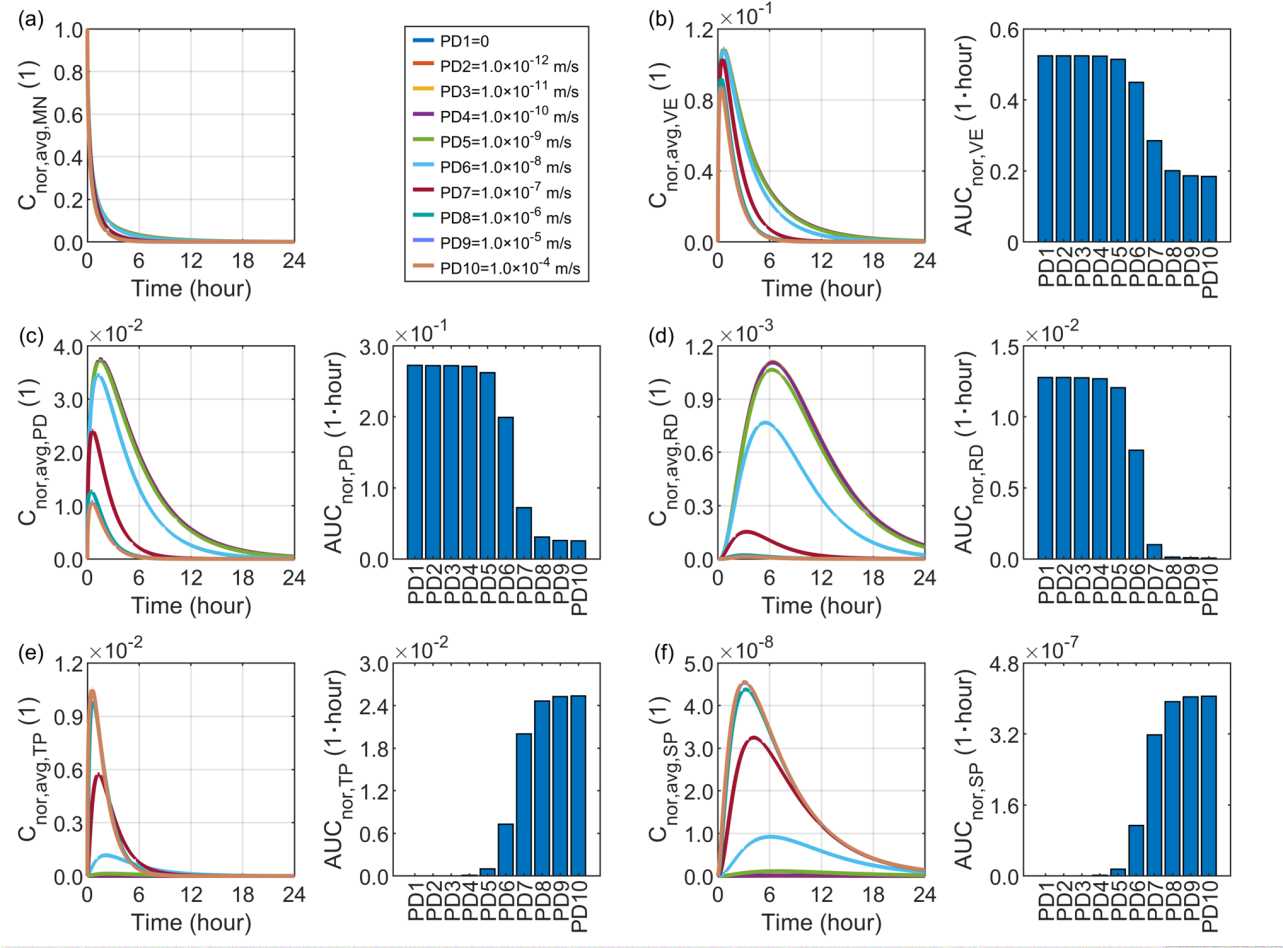
Delivery outcomes for drugs with different permeabilities to cross capillary walls. Subplot (**a**) shows the model-predicted drug concentration in the microneedle, while subplots (**b**) to (**f**) present drug concentration and exposure in the viable epidermis, papillary dermis, reticular dermis, tissue blood, and systemic blood, respectively.

The predicted delivery outcomes in Fig. 12 show that increasing the drug transvascular permeability from 0 to $1.0\times {10}^{-10} \mathrm{m}/\mathrm{s}$ has a negligible effect on drug accumulation and exposure in the microneedle and all skin layers, while the drug concentration in the blood compartments remain extremely low. When the permeability increases to $1.0\times {10}^{-6} \mathrm{m}/\mathrm{s}$, the drug availability in both the tissue blood and systemic blood rises sharply, whereas the drug concentrations in the microneedle and skin tissues decrease as more drug molecules penetrate into the bloodstream. Further increasing the permeability to $1.0\times {10}^{-4} \mathrm{m}/\mathrm{s}$ yields only a marginal improvement in the plasma drug concentration; the impact on the delivery to the local tissues is also limited.

### Effect of Plasma Clearance Rate

**Fig. 13:**
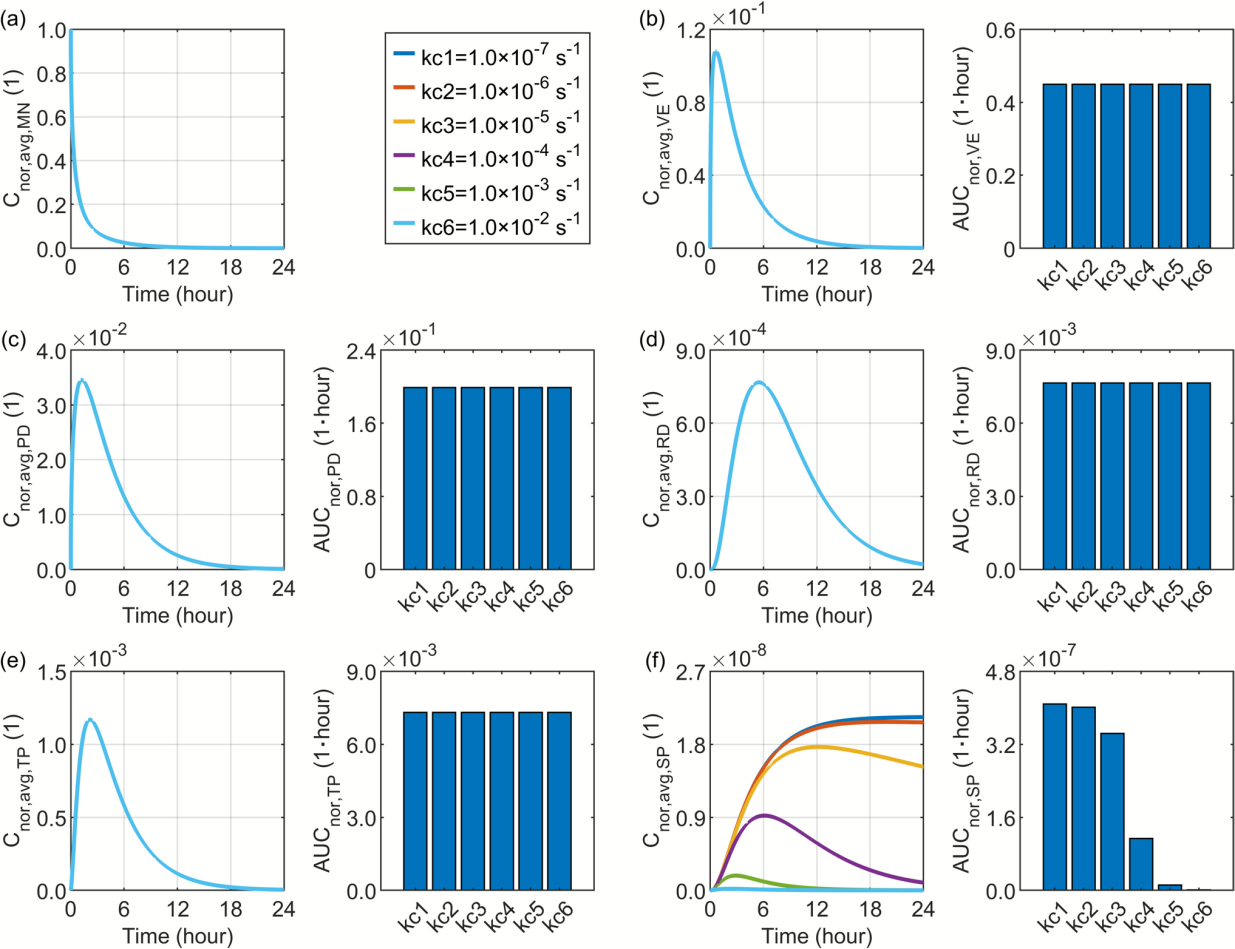
Delivery outcomes for drugs with different plasma clearance rates. Subplot (**a**) shows the model-predicted drug concentration in the microneedle, while subplots (**b**) to (**f**) present drug concentration and exposure in the viable epidermis, papillary dermis, reticular dermis, tissue blood, and systemic blood, respectively.

Drugs distributed in the systemic blood are eliminated primarily through organ-mediated clearance, predominantly by the liver and kidney. The characteristic timescale of this elimination process is represented by the plasma clearance rate, which varies substantially across different drug types. In this parametric analysis, a range of $1.0\times {10}^{-7} {\mathrm{s}}^{-1}$ to $1.0\times {10}^{-2} {\mathrm{s}}^{-1}$ is employed to evaluate its influence on the transdermal drug delivery outcomes, as illustrated in Fig. 13. The simulation results reveal that an increased plasma clearance rate greatly reduces the drug concentration in the systemic blood owing to enhanced elimination. Nevertheless, this effect remains largely confined to the systemic compartment, with negligible influence on the drug concentration and exposure within the skin layers and tissue blood.

### Comparisons Among Drugs

**Fig. 14:**
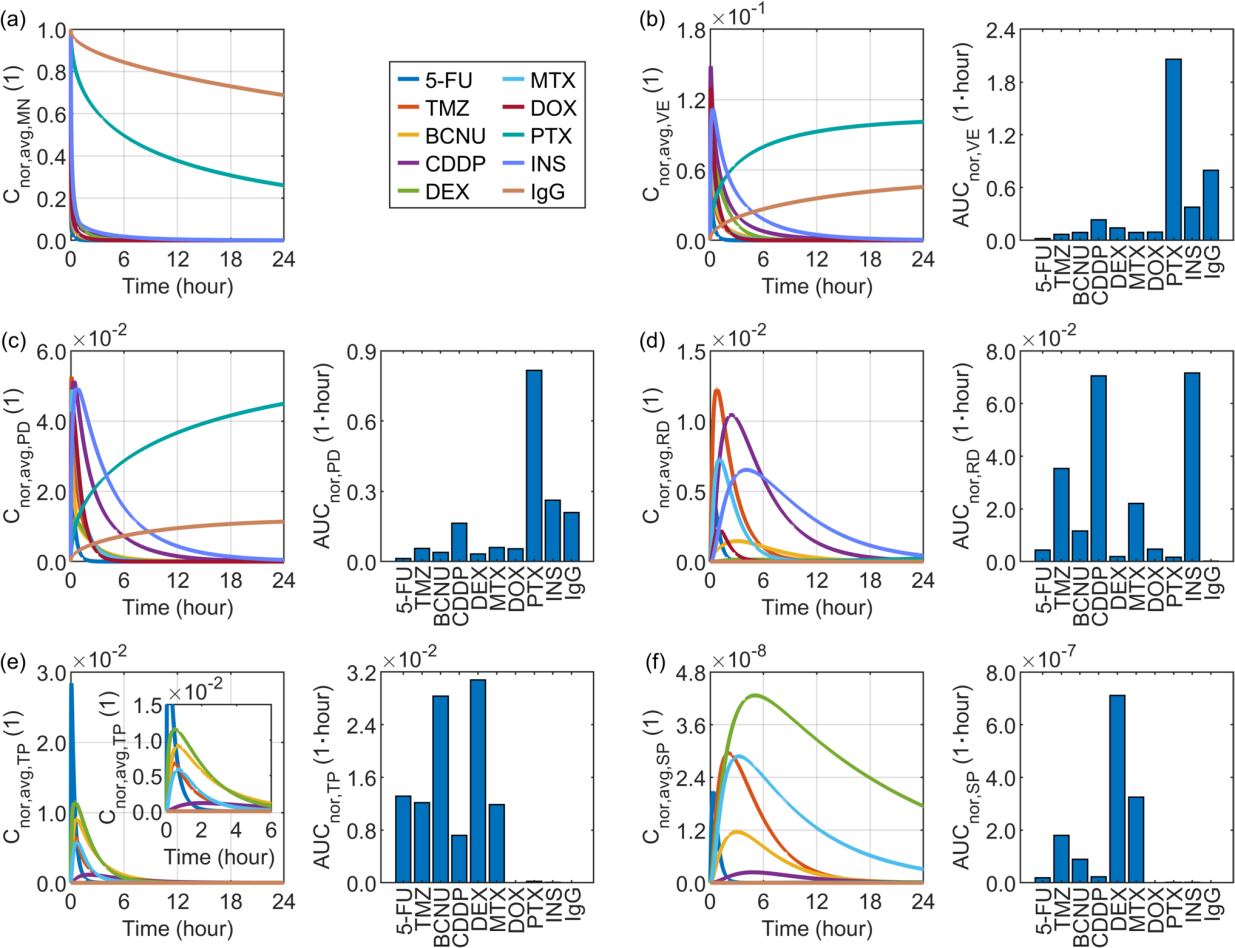
Modelling predicted delivery outcomes for various drugs. Subplot (**a**) shows the model-predicted drug concentration in the microneedle, while subplots (**b**) to (**f**) present drug concentration and exposure in the viable epidermis, papillary dermis, reticular dermis, tissue blood, and systemic blood, respectively.

Simulations are conducted to predict the delivery outcomes of the ten drugs, based on their specific transport properties summarised in Table II. The results in Fig. 14 indicate that both PTX and IgG exhibit sustained release from the microneedle, which can be attributed to their relatively low diffusivity and low elimination rates. The former reflects the slow molecular mobility of these drugs, whereas the latter leads to elevated local concentrations in the tissues adjacent to the microneedle, including the viable epidermis and papillary dermis. This high accumulation in the tissues reduces the concentration gradient across the microneedle–tissue interface, thereby retarding the drug release from the microneedle. However, these transport characteristics substantially limit their penetration into deeper tissue of reticular dermis and the systemic circulation, where the drug concentrations remain low. In contrast, although INS also possesses low diffusivity that may retard its transport, its relatively higher elimination rate leads to lower tissue concentrations, thereby establishing a steeper concentration gradient across the microneedle-tissue interface. This gradient facilitates faster release of INS from the microneedle. Furthermore, due to its low transvascular permeability, INS can more effectively traverse the papillary dermis and accumulate within the reticular dermis. Similarly, owing to their low transvascular permeability and high diffusivity, CDDP and TMZ also tend to accumulate effectively within the reticular dermis rather than in the upstream regions, including the papillary dermis and viable epidermis. Although BCNU exhibits comparable diffusivity and elimination rate, its high transvascular permeability enables greater penetration into the bloodstream. Nevertheless, its rapid plasma clearance results in a low systemic blood concentration. A similar trend is observed for 5-FU. In contrast, DEX achieves the most efficient delivery to the systemic circulation. This is attributed not only to its high transvascular permeability, which facilitates entry into the bloodstream, but also to its low plasma clearance, which prolongs its persistence in the circulatory system.

## Discussion

Microneedles can effectively deliver encapsulated drugs into viable skin tissues by penetrating the stratum corneum. Modelling results indicate that, upon application, the drug begins to diffuse from the microneedle into the surrounding tissues and gradually migrates towards the deeper skin layers and the blood capillaries located within the papillary dermis. As the drug concentration within the microneedle progressively decreases, the concentrations in the skin tissues and blood initially increase and subsequently decline over time. The concentration profiles across the skin layers and blood follow the same order as the drug transport pathway.

The delivery outcomes in different skin layers and the blood circulatory system exhibit distinct responses to variations in drug transport properties. Such non-uniformity highlights the potential for rational drug design or selection to not only enhance treatment efficacy at specific target sites but also to minimise adverse effects in non-target regions. For instance, drugs with higher diffusivity within the microneedle can effectively increase the peak concentration and overall exposure in most skin layers and the blood, except for the viable epidermis, where an optimal diffusivity value yields the best outcome. In contrast, an optimal diffusivity within the tissue is required to maximise both the peak concentration and exposure in most compartments, except in the reticular dermis, where higher tissue diffusivity results in more effective delivery. Drugs with an increased protein binding coefficient present higher drug retention in the viable epidermis but reduced delivery to the bloodstream. To achieve optimal delivery in the papillary and reticular dermis, intermediate values of this coefficient are preferable. Similarly, a drug with a higher partition coefficient between the tissue and microneedle presents better delivery to the viable epidermis, whereas an optimal value of this parameter is needed to maximise drug exposure or peak concentration across all layers. Conversely, a lower partition coefficient between the intracellular and interstitial spaces favours delivery to the reticular dermis and the bloodstream, while an optimal value benefits delivery to the viable epidermis and papillary dermis. Distinctly, the cell membrane-interstitial space partition coefficient between exhibits an optimal value in each skin layer and the blood compartments to improve local delivery efficiency. Furthermore, using drugs with a higher transvascular permeability enhances systemic drug availability, although drug retention in the skin is simultaneously reduced. Systemic availability can also be improved by selecting the drugs with lower plasma clearance, whereas this parameter shows negligible influence on drug concentrations within the skin. In addition, the drugs with a lower elimination rate are preferred, since higher elimination significantly reduces drug delivery efficiency across all skin layers and the bloodstream. It is also worth noting that the present parametric analyses are conducted to examine the influence of each drug transport property individually. Although optimisation of a single parameter is feasible, for instance, fine-tuning the drug diffusivity in the microneedle through the modification of microneedle formulation, multiple properties may vary simultaneously in practice. For example, drugs with smaller molecular weights often exhibit lower diffusivity in both the microneedle and the skin tissues. Therefore, investigating the combined effects of multiple parameters or quantifying the relative contribution of each property to the overall delivery outcomes would be highly beneficial. However, this aspect lies beyond the current study and is thus not addressed here.

**Fig. 15:**
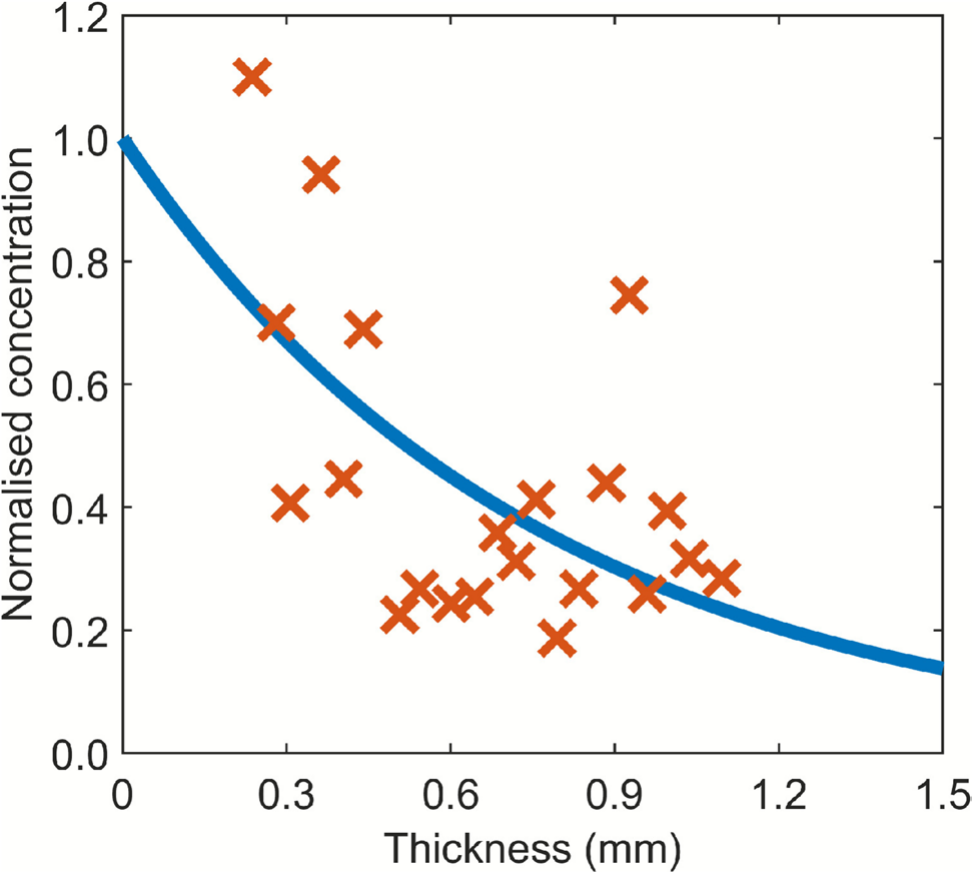
Comparison between the model-predicted spatial distribution of retinoic acid concentration in skin tissues and the corresponding experimental measurements. The experimental data and model parameters are extracted from the reference [70].

The model developed in this study aims to capture the principal drug delivery processes. The predicted concentration profile is compared with the reported experimental data in Fig. 15, showing good agreement and thereby validating the accuracy of the model. Similar validation studies have also been reported in previous works [70, 71]. Nevertheless, it is important to recognise that modelling studies primarily provide quantitative predictions. Although such results are adequate for assessing the influence of key factors through comparative analysis, they may not fully reproduce the *in vivo* conditions, which are highly variable, patient-specific, and dynamic. Moreover, it remains challenging to account for all delivery processes. To further improve the model, more experimental measurements of tissue properties and the development of real-time monitoring techniques would be valuable for better characterisation of the tissue microenvironment. *In vivo* experiments can also contribute to a deeper understanding of the detailed delivery mechanisms and guide model refinement. On the other hand, incorporating all such complexities would substantially increase computational demands, potentially making simulations computationally expensive or impractical. Therefore, appropriate simplifications are necessary to maintain a balance between model accuracy and computational feasibility [72].

This study also involves several limitations and assumptions. (1) Scope of the study. Only the effects of drug transport properties are investigated. In practice, the delivery outcomes are also influenced by multiple factors, including the type, geometry and formulation of the microneedles, their spatial arrangement on the supporting patch, the delivery regimen, various tissue characteristics, and environmental conditions such as temperature, wind speed, and relative humidity [71, 73–76]. The effects of these factors could be explored in future studies. However, it should be noted that these factors may interact in a cascading manner, leading to coupled changes in several parameters. For instance, tissue temperature not only affects the viscosity of the interstitial fluid but also determines the diffusivity in both the microneedle and the skin tissues, as well as the blood perfusion rate that links the tissue blood to the systemic circulation. Employing appropriate mathematical models to accurately capture these cross-dependencies will be crucial for future simulations. (2) Tissue deformation. The insertion of microneedles can mechanically compress the surrounding skin tissues, thereby reducing the volume fraction of the interstitial space through which fluid and drug transport occur. However, it remains unclear whether such deformation persists or relaxes during the long-term delivery process [77]. Consequently, this effect is not considered in the current model. A tissue mechanics module could be incorporated into the present framework to simulate the delivery process under tissue deformation [78]. Nevertheless, modelling potential tissue relaxation remains a major challenge [77]. (3) Evaluation of delivery outcomes. The present model evaluates delivery performance primarily in terms of drug transport and accumulation. In practice, the outcomes should also be assessed using more clinically relevant or qualitative indices, such as the local therapeutic efficacy, systemic bioavailability, or therapeutic index, depending on the pharmacodynamics of each drug. To further advance the model, drug-specific pharmacodynamic modules could be integrated to link the predicted concentration distributions with pharmacological responses. (4) Modelled delivery process. The terminal compartment in the current model is the systemic blood, rather than a specific target tissue, as in many therapeutic applications of transdermal delivery, such as the brain in the treatment of Alzheimer’s disease. Future studies focusing on particular disorders could incorporate the target tissue containing the lesions as the terminal compartment to provide a more comprehensive simulation of the overall therapeutic process.

## Conclusions

The dependence of microneedle-mediated transdermal delivery on the transport properties of the loaded drug is investigated through numerical simulations. The results reveal distinct responses of the delivery outcomes in each skin layer and in the systemic blood to variations in the drug properties. Specifically, drugs with higher diffusivity in the microneedle or a higher tissue-microneedle partition coefficient produce a faster decline of drug concentration in all skin layers and in the blood. The former enhances drug exposure in all compartments except the viable epidermis, where this parameter requires optimisation, whereas the latter increases drug exposure in the viable epidermis but requires optimisation in other skin layers and the blood. Optimal values are also identified for the diffusivity in the skin tissues and the cell membrane-interstitial space partition coefficient, depending on the location of the targeted site within the skin. Similarly, the protein-binding coefficient exhibits optimal values for drug delivery to different skin layers, while drug delivery to the blood decreases as this coefficient increases. A higher partition coefficient between the intracellular and interstitial spaces results in lower peak concentrations but more sustained levels in the viable epidermis and papillary dermis; in these layers, optimal values of this parameter can improve overall exposure. Conversely, drug delivery to the reticular dermis and the blood is reduced when this parameter is high. Furthermore, delivery to the systemic circulation can be enhanced by using drugs with higher transvascular permeability or a lower plasma clearance rate. The former, however, reduces drug concentrations within the skin layers, while the latter shows limited influence on drug distribution in the skin. Drugs with a lower elimination rate improve delivery in both the skin layers and the blood. Overall, the findings from this comprehensive parametric study provide insights into optimising microneedle-mediated transdermal delivery. They can inform drug selection and design from the perspective of drug transport and accumulation, thereby facilitating more effective and predictable transdermal therapeutic strategies.

## Data Availability

The datasets generated and/or analysed during the current study are available from the corresponding author upon reasonable request.
